# *In Vitro* Study for Combating Multidrug-Resistant Pathogens via a Facile Sustained Release of Benzoic Acid and Parabens from PMMA/PCL Nanofibrous Membrane

**DOI:** 10.3390/pathogens15090880

**Published:** 2026-08-22

**Authors:** Reham M. Goda, Alaa A. Omar, Ibrahim A. Maghrabi, Mohamed F. El-Badawy, Islam M. Bendary, Mohamed M. Shohayeb, Mohamed Abd El-Gawad El-Sayed Ahmed

**Affiliations:** 1 Department of Microbiology and Immunology, Faculty of Pharmacy, Delta University for Science and Technology, Gamasa 11152, Egypt; reham.gouda@deltauniv.edu.eg (R.M.G.); mohamed.shohayeb@deltauniv.edu.eg (M.M.S.); 2 Nanomedicine Research Unit, Delta University for Science and Technology, Gamasa 11152, Egypt; alaa.bokharist@deltauniv.edu.eg; 3 Department of Clinical Pharmacy, College of Pharmacy, Taif University, Taif 21944, Saudi Arabia; i.maghrabi@tu.edu.sa; 4 Department of Microbiology and Immunology, Faculty of Pharmacy, University of Sadat City, Sadat City 32897, Egypt; 5 Department of Dental Biomaterials, Faculty of Dentistry, Delta University for Science and Technology, Gamasa 11152, Egypt; islam.mahmoud@deltauniv.edu.eg; 6 Department of Microbiology and Immunology, Faculty of Pharmaceutical Sciences and Drug Manufacturing, Misr University for Science and Technology (MUST), Giza P.O. Box 77, Egypt; mohamed.gawad@must.edu.eg

**Keywords:** multidrug resistance, MRSA, benzoic acid, electrospun nanofibrous membranes, wound dressings

## Abstract

The global threat of multidrug-resistant infections requires new approaches for its management. A blend of polymethyl methacrylate (PMMA) and poly(ε-caprolactone) (PCL) electrospun nanofibrous membrane was utilised as a scaffold to sustain the release of three broad-spectrum antiseptics to combat multidrug-resistant (MDR) microorganisms. The scaffold was characterised by Fourier transform infrared spectroscopy, contact angle, and scanning electron microscopy. The latter revealed a random and smooth structure. The contact angle value of 81.8 ± 1.8 confirmed the hydrophilic nature of the scaffold, which is important for wound healing. The high surface-to-volume ratio of the scaffolds was utilised for loading benzoic acid (BA), methylparaben (MPB), and propylparaben (PPB) which were released during 72 h concentrations ranging between 0.20 and 0.8 mg mL^−1^. The initial release of antiseptics was high and was then sustained for over 72 h. After 8 h, the burst release was 61.64 ± 4.11% for BA, 41.11 ± 2.98% for MPB and 34.32 ± 3.13% for PPB. The release profile of BA was superior to that of the MPB and PPB. The loaded scaffolds inhibited MDR-resistant methicillin-resistant *Staphylococcus aureus*, *Escherichia coli* and *Candida albicans* and were not cytotoxic to a fibroblast cell line. They inhibited their colonisation by the tested microorganisms to non-detectable counts or at least reduced microbial counts by at least 6–7 logs (*p* ≤ 0.001) for 72 h. Crystal violet techniques and electron microscopy confirmed colonisation inhibition. Because of their non-cytotoxicity and broad-spectrum antimicrobial activity, the antiseptic-loaded PMMA/PCL nanofibrous scaffolds could be utilised as wound dressings, particularly in non-healing wounds.

## 1. Introduction

Antibiotic resistance is one of the greatest threats to global health systems [1]. The misuse of antibiotics results in the development of resistant bacterial strains. In 2019, over 1.27 million people died because of infections not responding to existing antibiotics. In the United States, at least 2 million people are infected with antibiotic-resistant bacterial strains every year [2]. Estimations expect that by 2050, the mortality rate will reach 4.73 million in Asia, 4.15 million in Africa, 0.39 million in Europe, 0.392 million in Latin America, and 0.317 million in North America [3]. The management of microbial infections requires an expenditure of millions of dollars.

Bacteria that cause wound infections are mostly multi-drug resistant (MDR). Therefore, to succeed in preventing infections, there is always a need for novel antibiotics [4]. Unfortunately, the research and development process to produce a new antibiotic is expensive and time-consuming [5,6]. Therefore, the discovery and development of genuinely new antibiotics is a big challenge. Therefore, there is an urgent need for alternative strategies to combat antibiotic-resistant strains [7]. Luckily, some antiseptics and preservatives are broad spectrum and effective against MDR bacteria. These compounds have several mechanisms of action, and therefore, the development of resistance for them is unlikely. Nonetheless, antiseptic dressings for preventing and managing biofilm and infection progression need further research [8].

Benzoic acid (BA), methylparaben (MPB), and propylparaben (PPB) have broad-spectrum antimicrobial properties as antiseptics and preservatives. They are widely used as preservatives in foods, pharmaceuticals, and mouthwashes [9]. Therefore, they may be successful against MDR bacterial strains [10].

Nanofibrous scaffolds are characterised by their high surface-area-to-volume ratio and high porosity. They can be fabricated from various materials, including biodegradable and non-biodegradable polymers [11]. Therefore, they have versatile applications. In the biomedical field, they can be used as biomaterials in various applications such as regenerative medicine and wound healing [12]. Researchers have already developed nanofibrous-based wound dressings to accelerate healing and tissue regeneration [13], where pharmaceutically formulated nanofibrous membrane have been used as carriers for controlled drug delivery [14].

Different techniques for nanofibrous scaffold fabrication are used where the most commonly used technique is electrospinning [15]. The electrospinning technique is one of the best options for production of nanofibrous membrane due to its flexibility, simplicity, and cost-effectiveness [16]. Nanometre-sized diameter fibres are deposited from a needle on a substrate [17,18]. A polymer’s structure and physical properties can be modified to specific requirements by blending two or more polymers [19]. Physical mixing of polymers results in novel properties superior to those of each individual polymer used alone [20].

Polycaprolactone (PCL) is one of the most promising biodegradable and biocompatible aliphatic polymers. It has been approved by the US Food and Drug Administration (FDA) for medical applications and drug delivery systems [21]. The blend of PMMA/PCL nanofibrous membrane, prepared by electrospinning, is characterised by its homogeneity, miscibility, and improved mechanical strength [22]. In the same context it is important to know that the control of loading efficiency and drug release profile reduces toxicity [23].

This study explored the use of electrospun PMMA/PCL nanofibrous membranes as a reservoir for the sustained release of BA, MPB, and PPB. The antimicrobial/antibiofilm effect of the loaded nanofibrous membrane was evaluated against *Staphylococcus aureus* (*S. aureus*), *Escherichia coli* (*E. coli*), and *Candida albicans* (*C. albicans*), as examples of pathogenic Gram-positive bacteria, Gram-negative bacteria, and a fungus, respectively. Nanofibrous membranes were also evaluated for their cytotoxicity on a fibroblast cell line.

## 2. Materials and Methods

### 2.1. Chemicals, Microbiological Media, and Antibiotics

Polycaprolactone (PCL, Mwt~80 kD) was purchased from Spectrum Chemical MFG. Corporation, New Brunswick, NJ, USA. Polymethyl methacrylate (PMMA, Mwt~120 kD) was purchased from Alfa Aesar, Mumbai, India. Dimethylformamide, dichloromethane, methanol and ethanol were obtained from Alpha Chemika, ndheri, Suresh Nagar, andheri West, Mumbai, India. Nutrient broth, mannitol salt agar, MacConkey’s agar, tryptone-soy broth (TSB), Mueller-Hinton agar(MHA), Mueller-Hinton broth (MHB), Sabouraud dextrose agar (SDA), Sabouraud dextrose broth (SDB) were purchased from Oxoid (Hampshire, UK). Dulbecco’s Modified Eagle Medium (DMEM) was purchased from Gibco (Thermo Fisher Scientific, Waltham, MA, USA). Human skin fibroblast (HSF) supplied from Nawah Scientific Inc., Mokatam, Cairo, Egypt. Oxacillin (5 µg), cefotaxime (30 µg), ceftriaxone (30 µg), cefoxitin (30 µg), ciprofloxacin (5 µg), tobramycin (10 µg), erythromycin (15 µg), doxycycline (30 µg), gentamycin (10 µg), sulfamethoxazole-trimethoprim (1.25/23.27 µg), imipenem (10 µg), amikacin (30 µg), aztreonam (30 µg) and vancomycin (30 µg), tetracycline (30 µg), fusidic acid (10 µg), amphotericin B (10 µg), and fluconazole (25 µg) were obtained from Oxoid, Hampshire, England.

### 2.2. Bacterial Strains

Clinical isolates of *S. aureus*, *E. coli* and *C. albicans* were obtained from the culture collection of the Microbiology and Immunology Department, Faculty of Pharmacy, Delta University for Science and Technology, Gamasa, Egypt. All clinical isolates involved in the current study were reconfirmed phenotypically by standard biochemical methods [24]. The isolates were kept at −80 °C in a TSB containing 20% glycerol for subsequent testing.

### 2.3. Preparation of the PMMA/PCL Blend Solution

Briefly, PMMA (4% *w*/*v*) and PCL solution (10% *w*/*v*) were separately dissolved in a mixture of dimethylformamide and dichloromethane with ratio (2:1) at room temperature. PMMA and PCL solutions were mixed in a ratio of 30:70 under magnetic stirring for 2 h at room temperature [25,26].

### 2.4. Fabrication of PMMA/PCL Nanofibrous Scaffold via Electrospinning Technique

The PMMA/PCL solution was loaded into a 10 mL syringe fitted with a 22-gauge needle, and the collector-needle distance was 15 cm. The needle was mounted into the electrospun nanofibrous system (NANON-01A, MECC, Ogori City, Fukuoka Prefecture, Japan) using a clip spinneret. The flow rate was 0.5 mL/h. The direct current (DC) positive voltage-power supply was 30 kV and the relative humidity was about 55% at room temperature. The developed electrospun nanofibrous membrane was collected on a sterile metallic paper, and air-dried. The irregular boundaries of the scaffold were cut with a sterile ruler and a sharp scalpel and the thickness of the membrane was measured by a digital calliper (electronic micrometre, Adoric, Denver, CO, USA) before storage between sterile papers for further use [25].

### 2.5. Loading the PMMA/PCL Scaffold with BA, MPB and PPB

BA, BA, MPB and PPB were dissolved separately in an ethanol/acetone (1:1, *v*/*v*) mixture at a typical concentration of 15 mg mL^−1^ and heated gently at 50 °C under stirring until fully dissolved. Square pieces (1 cm × 1 cm) of the 0.35 mm thick sterile PMMA/PCL scaffold were cut aseptically, and each strip was weighed (*W_i_*). The pieces were dipped separately into the loading solution (15 mg mL^−1^) of either BA, MPB, or PPB and incubated with mild shaking (100 rpm) for 6 h at room temperature. The strips were taken out, gently blotted with filter paper to remove excess solvent, air-dried overnight at room temperature in a sterile glass Petri dish, and the final weight (*W_f_*) was determined. The loading efficiency of either BA, MPB, or PPB was calculated relative to the total weight of the loaded scaffold (*W_f_*). The loading efficiency and loading capacity were calculated according to the following Equations (1) and (2), respectively:

(1) $$Loading\;efficiency\;(\%)=\frac{W_{f}-W_{i}}{W_{i}}\times 100$$

(2) $$Loading\;capacity\;\left(\%\right)=\frac{W_{f}-W_{i}}{W_{t}}\times 100$$

where $W_{i}$, $W_{f}$ and *W_t_* represented the initial weight, the final weight and the total weight of scaffolds, respectively.

### 2.6. Scanning Electron Microscope (SEM) of Nanofibrous Membranes

The surface morphology of the nanofibrous membranes was characterised by a scanning electron microscope (SEM, JSM-6510 LV, JEOL (Peabody, MA, USA), USA). The nanofibrous scaffolds were coated using gold sputtering before imaging. Nanofibre diameters were calculated as the average diameter of nanofibrous scaffolds at different positions in SEM images using the ImageJ2 software program.

### 2.7. Fourier Transform Infrared Spectroscopy/Attenuated Total Reflectance (FTIR-ATR) of Nanofibrous Membranes

The chemical composition of the electrospun nanofibrous membranes was analysed by FTIR (Jasco 2100, Easton, MD 21601, USA) coupled with Attenuated Total Reflectance (ATR) mode to obtain a typical peak associated with PMMA and PCL. The peaks were recorded at a wavenumber range from 4000 to 400 cm^−1^.

### 2.8. X-Ray Diffraction (XRD) of Nanofibrous Membranes

The Philips PW-1729 X-ray diffraction spectrometer (Eindhoven, Netherlands) was used to measure the diffraction pattern of the fibrous nanofilms (20 mm × 20 mm × 0.35 mm) at angles ranging from 4° to 80°. To study the crystallinity of the films, Cu Kα radiation was used at a current of 200 mA, a scan rate of 4 °C/min, and an acceleration voltage of 45 kV.

### 2.9. Differential Scanning Calorimetry (DSC) of Nanofibrous Membranes

To investigate the thermal stabilities of the nanofibrous membranes, DSC tests were performed using a DSC TA (Q200, TA Instruments Inc., New Castle, DE, USA). The following heating scans, in a nitrogen flow at a heating rate of 10 °C/min, were performed from 25 to 800 °C. The thermal parameters were evaluated both during the first scan, representative of the as-prepared samples, and the second scan. An empty hermetic pan served as a baseline, and nanofibrous membranes (5 mg) were hermetically sealed in an aluminium pan. The DSC thermograms were used to determine the thermal characteristics of nanofibrous membranes, including T_m_ and H_m_.

The degree of crystallinity (x%) of the composites was estimated using the following Equation (3):

(3) $$x(\%)=\frac{∆H}{{\left(1-m\right)\;∆H}_{m\;\;}^{0}}$$

where $\left(1-m\right)\;$ is the nominal weight fraction of PCL (0.93), ∆H is the measured enthalpy for the melting process of PCL and ${∆H}_{m\;}^{0}$ = 135 − 136.1 (J/g) is the melting enthalpy for 100% crystalline PCL [27].

### 2.10. Thermogravimetric Analysis (TGA) of Nanofibrous Membranes

Thermal stabilities of the nanofibrous membranes were conducted with a thermogravimetric analyser (Q500; TA Instruments Inc., New Castle, DE, USA). For three minutes, nanofibrous pieces were kept at an isothermal temperature after being heated from 25 to 800 °C. Under a nitrogen atmosphere, the temperature was increased at a steady rate of 10 °C/min. Weight loss versus temperature was used to depict the data.

### 2.11. Tensile Strength of Nanofibrous Membranes

A universal material testing machine (Instron 3345, High Wycombe, Buckinghamshire, UK) was used to determine the mechanical properties of the nanofibrous membranes according to ASTM D882-02 standard methods [28]. The initial distance between the grips was 20 mm, and the tensile speed was 5 mm/min. The specimen was cut to the size of 40 mm × 10 mm × 0.35 mm and placed between the grip heads of the machine to be measured. The initial gauge length and grip separation were 50 mm. Each sample was analysed at least three times.

### 2.12. Hydrolytic Degradation of Nanofibrous Membranes

Hydrolytic degradation of the nanofibrous scaffold was assessed in simulated body fluid (SBF, pH 7.4). Square-shaped *W_i_*-scaffolds (2 cm × 2 cm × 0.35 mm) were immersed in 50 mL SBF at 37 °C at 150 rpm. At specific time intervals (1 to 20 days), the pieces were removed, washed in distilled water, blotted with soft paper and dried at 50 °C until a constant weight (*W_f_*). The media were changed and replaced with fresh media every day. The experiment was done in triplicate. Degradation (% weight loss) was calculated using the following Equation (4):

(4) $$Degradation\;(Weight\;loss)\;\%=\frac{W_{i}-W_{f}}{W_{i}}\times 100$$

where *W_i_* is the initial weight and *W_f_* is the final dry weight of the sample each day.

### 2.13. Swelling Behaviour (Water Intake) of Nanofibrous Membranes

Briefly, pieces of dry-weighted nanofibrous membranes (*W_d_*) were soaked in simulated body fluid (pH 7.4), acidic medium (pH 4), and ethanol (70%) in a shaking incubator at 37 °C at 150 rpm. At time intervals (up to 7 h), the pieces were removed from the media, and the surface water was removed using tissue paper for a few seconds for final weighing (*W_s_*) to determine the weight change of the sample over time. The experiment was done in triplicate. The swelling was calculated using the following Equation (5):

(5) $$Swelling\;ratio\;(\%)=\frac{W_{s}-W_{d}}{W_{d}}\times 100$$

where *W_d_* is the initial dry weight and *W_s_* is the swollen weight of the sample each day.

### 2.14. Porosity of Nanofibrous Membranes

A known volume of 100% ethanol bath was used to calculate the porosity of the nanofibrous membranes, as described by Ho and Hutmacher (2006) [29]. The experiment was done in triplicate. The porosity was calculated using the following Equation (6):

(6) $$Porosity\;(\%)=\frac{V1-V3}{V2-V3}\times 100$$

where *V1* is the initial known volume of 100% ethanol, *V2* is the volume sum of ethanol and submerged membrane, and *V3* is the remaining volume of ethanol in the bath after removing the membrane.

### 2.15. The Wettability of Nanofibrous Membranes

The wettability (contact angle) of the electrospun nanofibrous membranes was evaluated using the water contact angle method (SL600, Solon Information Technology Co., Inc., Solon, OH, USA). Briefly, a 4 µL drop of distilled water was placed on the surface of the nanofibrous membrane in air, and the contact angle was recorded using a densitometer. The droplet was photographed at 10 Hz for 10 s with a professional camera, and a static image perpendicular to the nanofibrous axis was used for analysis. Measurements were performed in triplicate, and the contact angle was determined by fitting the droplet contour with a Young–Laplace surface [30,31,32].

### 2.16. In Vitro Antiseptics-Release Profile from Nanofibrous Membranes

Standard calibration curves of BA, MPB and PPB in ethanol/acetone (1:1) were prepared. The release profile of the antimicrobial agents was carried out for 72 h. Nanofibrous pieces (1 cm × 1 cm × 0.35 mm) were impregnated in 5 mL of phosphate-buffered saline (PBS, pH 7.4). At time intervals, 1 mL from the stock vial was drawn and placed in a clean quartz cuvette, and the absorbance was measured at 230 and 254 nm for BA and parabens, respectively, using a UV–Vis spectrophotometer (Shimadzu spectrophotometer, model UV-900i, Tokyo, Japan). Then 1 mL of PBS was added back to the impregnation solution. The concentration of the released drug was calculated from the standard curve, and the cumulative drug release was determined [33].

(7) $$Cumulative\;release\;\left(\%\right)=\frac{M_{t}}{M\infty }\times 100$$

where $M_{t}$ was the amount released at time *t* and *M* was the total loaded drug.

### 2.17. Cytotoxicity Assay of the Nanofibrous Membranes

Cell viability was assessed by Sulforhodamine B (SRB) assay. Briefly, 1.5 cm^2^ from each membrane containing BA, MPB and PPB was cut, and placed in the 24-well plate for subsequent cell seeding. HSF were maintained in DMEM media supplemented with 100 mg mL^−1^ of streptomycin, 100 units/mL of penicillin and 10% of heat-inactivated foetal bovine serum in a humidified, 5% (*v*/*v*) CO_2_ atmosphere at 37 °C. The cytotoxicity assay was assessed as previously described [34]. Aliquots of 100 μL cell suspension (5 × 10^3^ cells) were loaded into a 96-well plate and incubated in the supplemented media, in addition to the control. After incubation for 24, 48 and 72 h, cells were fixed by replacing the medium with 150 μL of 10% trichloroacetic acid (TCA) and refrigerating at 4 °C for 1 h. The TCA solution was removed, and the cells were washed 5 times with distilled water. Aliquots of 70 μL of 0.4% *w*/*v* sulforhodamine B solution were added to each well, and the plate was incubated in a dark place at room temperature for 10 min. The wells of the plate were washed three times with 1% acetic acid, drained and allowed to air-dry overnight. Then, 150 μL of 10 mM TRIS buffer, pH 7.5, was added to each well to dissolve the protein-bound SRB stain, and the absorbance was measured at 540 nm using a microplate reader (Tecan, Männedorf, Zurich, Switzerland) [34].

### 2.18. Screening of mecA Gene Among S. aureus Clinical Isolates by Polymerase Chain Reaction (PCR)

DNA was extracted from the MRSA isolates by the boiling method [35] where the *mecA* gene was investigated as described before [36] using the following primer sequences, Fw: AAA ATC GAT GGT AAA GGT TGG C, and Rv: AGT TCT GCA GTA CCG GAT TTG C, and the following cycle conditions, initial denaturation was at 94 °C for 30 s, the annealing was at 55 °C for 30 s, and the extension was at 72 °C.

### 2.19. Determination of Antimicrobial Susceptibility by the Diffusion Method

Antibiotic susceptibility testing was performed using the disc diffusion method as previously described [37] where the results were interpreted according to CLSI guidelines [38]. Briefly, bacterial suspension of the tested isolate was prepared and adjusted to 0.5 McFarland turbidity standard. A sterile cotton swab was dipped into the microbial suspension before streaking bacteria onto the surface of a dried MHA plate. In the case of *C. albicans*, SDAwas used. The inoculated plates were left undisturbed for 3–5 min on a flat surface. After that, either antibiotic discs or nanofibrous pieces (0.5 cm^2^) loaded or unloaded with antiseptics were placed onto the inoculated plates. Plates were examined for the formation of inhibition zones after 18 h incubation at 37 °C in the case of bacteria or 25 °C in the case of *C. albicans*.

### 2.20. Determination of the Minimal Inhibitory Concentration (MIC) of BA, MPB and PPB

The MIC was performed by the broth microdilution method, according to the CLSI guidelines [38]. Briefly, bacterial turbidity was adjusted to a 0.5 McFarland standard. Antiseptics were twofold serially diluted in microtiter plates in MHB for bacteria and in SDB for *C. albicans*. Wells were inoculated with microorganisms and incubated for 18 h for bacteria and 48 h for the *C. albicans*. The MIC was calculated as the lowest concentration of the antibiotic that completely inhibited the growth of the tested organism.

### 2.21. Determination of Microbial Viable Counts in Biofilms Formed on PMMA/PCL Nanofibrous Membranes

Each piece of the antiseptic-loaded or unloaded PMMA/PCL membrane was immersed in a test tube containing 5 mL of nutrient broth containing 10^5^ CFU mL^−1^ of the tested microorganism. The tubes were incubated at 37 °C for 24 h. Membrane pieces were removed, washed three times with sterile distilled water, then placed in 5 mL sterile saline, and exposed to a sonication bath for 5 min (MCS Digital ultrasonic cleaner, Osaka, Japan). The detached bacteria released from the strips were serially diluted, plated, and incubated for 24 h at 37 °C for bacteria and 48 h for *C. albicans*. Colonies representing the organisms colonising the strips were counted. Positive and negative controls were included in all experiments [39].

### 2.22. Examination of Biofilm Formation by Scanning Electron Microscopy (SEM)

The adherence of the tested bacteria and *C. albicans* on its surface with or without antiseptic-loading with BA, MPB, and PPB. The pieces of membranes were washed with PBS and fixed with 2% glutaraldehyde for 2 h. After dehydration with ethanol, the pieces of membranes were sputter-coated with Au–Pd (60:40 ratio) and checked using scanning electron microscopy (SEM, JSM-6510 LV, JEOL, USA) [40].

### 2.23. Examination of Microbial Biofilm Formation by the Crystal Violet Method

Bacteria were grown in MHB at 37 °C for 24 h, and *C. albicans* was grown for 48 h in SDB. Samples from each microorganism were collected, washed, suspended in saline, and the turbidity adjusted to 0.5 McFarland standard. Eppendorf tubes were filled with 1 mL MHB for bacteria or SDB) for the *C. albicans*, supplemented with 1% glucose. The tubes were inoculated with 20 µL of the tested microorganisms. To each tube a piece of nanofibrous membrane (0.5 cm^2^) was added. Each piece was loaded with one of the tested antiseptics. Unloaded pieces of nanofibrous membranes were included and acted as controls. After incubation for 48 h at 37 °C and 25 °C for the tested bacteria and *C. albicans*, respectively, nanofibrous membranes were removed and washed three times with sterile PBS (pH 7.2) and each impregnated in 1mL methanol for 20 min, to fix the adherent microorganisms. The pieces were then left at room temperature for 18 h air-drying. All nanofibrous membranes were stained, each in an Eppendorf tube containing Gram-staining crystal violet for 15 min at room temperature, and then rinsed with running tap water until the washings were dye-free. The dye acquired by each nanofibrous membrane was solubilised in 200 µL 95% alcohol, and the absorbance of the released crystal violet from each nanofibrous membrane was measured at 630 nm using a Sunrise Microplate Reader (Tecan, Segrate (Milan), Italy).

### 2.24. Statistical Analysis

Except for DSC, TGA, and XRD analysis, which were performed once, all experiments were repeated at least three times as mean ± standard deviation (±SD). Data analyses were carried out using the Statistical Package for the Social Sciences (SPSS, software version 20, 2017, IBM Corporation, North Castle, NY, USA). The significant differences (*p* ≤ 0.05) between result values in the samples in the measurements of the thermal properties (Tm), mechanical properties (tensile strength and elongation at break), antimicrobial activities, biodegradability, swelling, porosity and *in vitro* cytotoxicity were identified by one-way analysis of variance (ANOVA) followed by Duncan’s multiple range tests.

## 3. Results

### 3.1. Morphology Investigation of PMMA/PCL Nanofibrous Membranes

**Figure 1:**
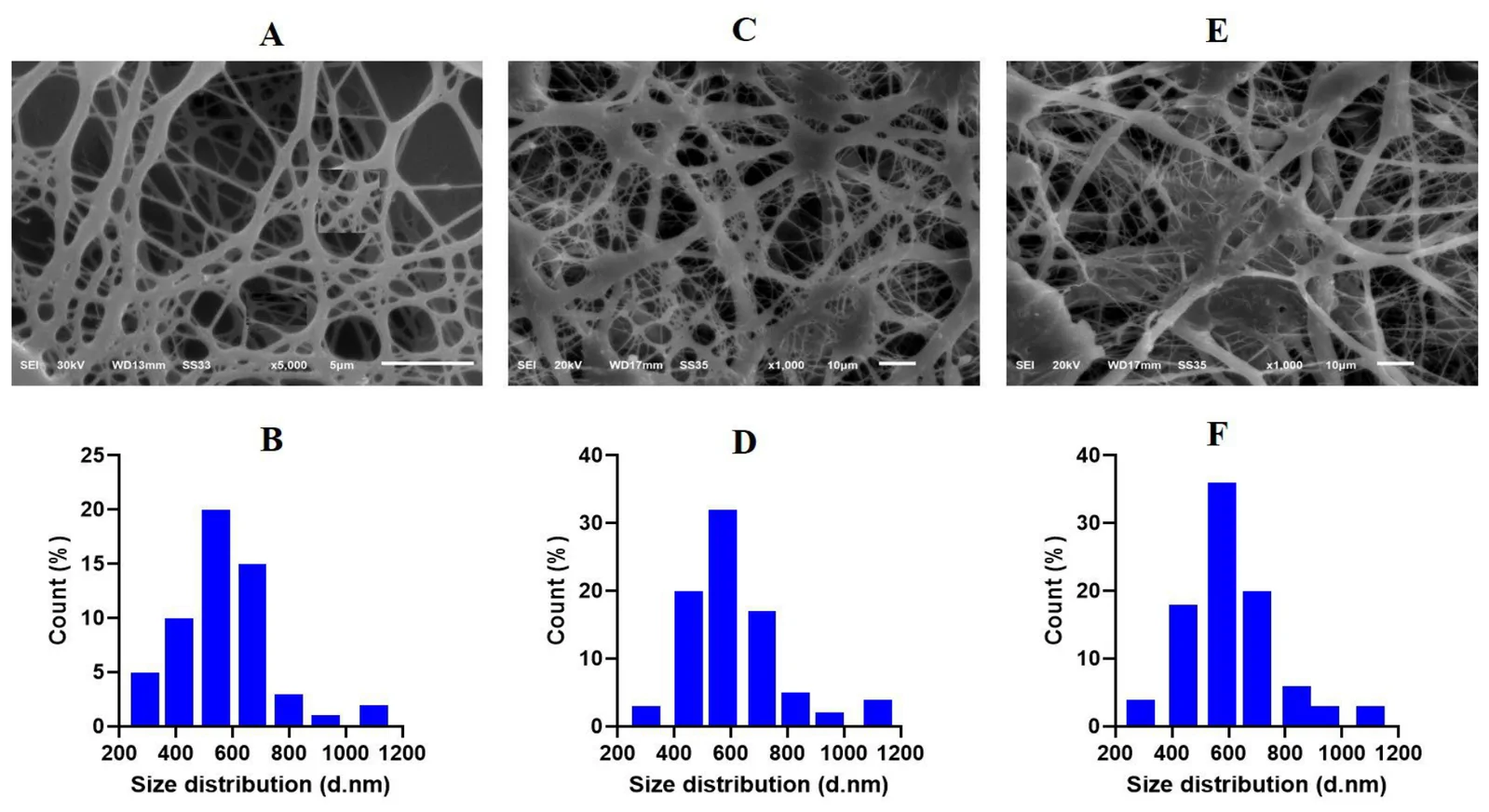
SEM image of pure PMMA/PCL (**A**), BA-loaded PMMA/PCL (**C**) and Parabens-loaded PMMA/PCL (**E**) electrospun nanofibrous scaffolds and their respective size distribution (**B**,**D**,**F**).

The electrospinning technique was used to produce sheaths of loaded and unloaded PMMA/PCL with dimensions of 7 cm × 5 cm and a thickness of about (0.35 ± 0.1) mm. Results showed droplet-free, non-woven, randomly orientated nanofibrous sheets. The nanofibres were arranged randomly with a smooth structure, as appeared under SEM (Figure 1). The average diameter of different scaffolds was calculated using ImageJ2 software by detecting and analysing fifty random nanofibres. The diameter of the different PMMA/PCL nanofibrous membranes ranged between (290 and 1100) nm, and the most predominant diameter was (571 ± 173) nm (Figure 1).

### 3.2. FTIR Analysis

**Figure 2:**
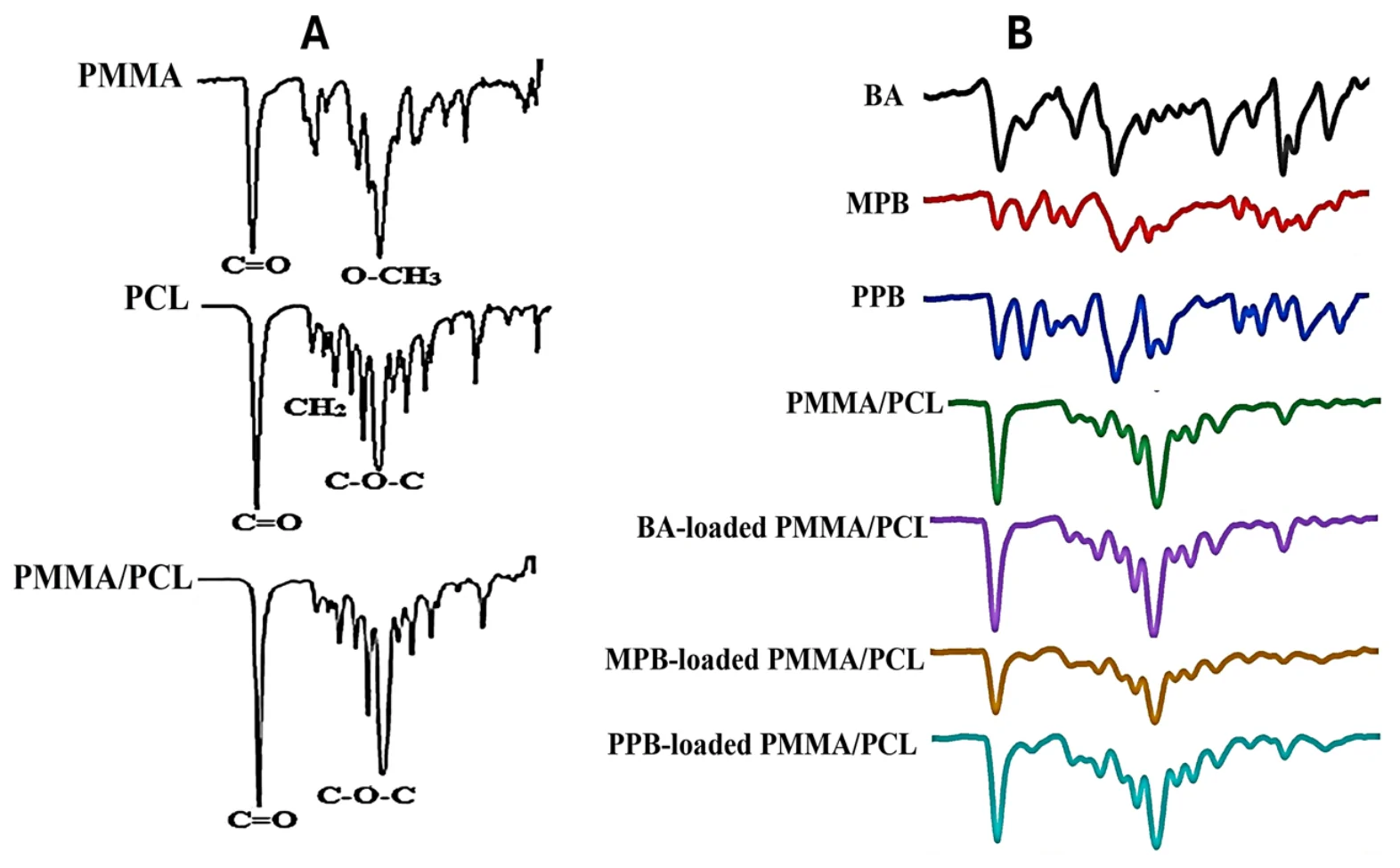
FTIR spectra of (**A**), PMMA, PCL and PMMA/PCL nanofibrous membrane; (**B**), benzoic acid (BA), methylparaben (MPB), Propylparaben (PPB), PMMA/PCL, BA-PMMA/PCL, MPB-loaded PMMA/PCL and PPB-loaded PMMA/PCL nanofibrous membrane.

FTIR absorption spectra revealed the physical incorporation of the PMMA/PCL blend in the nanofibrous membranes and physicochemical interaction between nanofibrous membranes and antiseptics (Figure 2). The specific absorption bands of PCL related to C=O, CH_2_ bending and C–O–C modes were detected at 1722.12, 1365.35, and 1178.29 cm^−1^, respectively. PMMA displayed characteristic absorption peaks at 1722.12 and 1240 cm^−1^, which are assigned to the stretching vibration of C=O bonds and O–CH_3_, respectively. The PMMA/PCL nanofibrous membranes exhibited peak shifts, disappearance, and new absorption peaks detected at around 1202.12 cm^−1^.

As shown in Figure 2B, the FTIR spectra of BA shows the acidic –OH stretching vibration as a broad peak from 3073.04 cm^−1^ to approximately 2883.05 cm^−1^ and the C=O stretching vibration at 1676.09 cm^−1^, while the peak of the aromatic C=C vibrational stretch is present around 1496.82 cm^−1^, and aromatic C–H out-of-plane bending at 742.24 cm^−1^. Other bands are also present which are characteristic of this aromatic carboxylic acid compound. The PMMA/PCL nanofibrous characteristic peaks do not alter when compared to BA and the blank PMMA/PCL nanofibrous membrane. The overlap between BA carboxylic C=O and polymer ester C=O causes the carbonyl band at 1722.99 cm^−1^ to somewhat widen. The 1467.20–1416.28 cm^−1^ area exhibits weak aromatic vibrations that can be attributed to BA. There are no fresh bands of absorption. Instead of being chemically linked, BA is physically enclosed within the PMMA/PCL nanofibrous, as indicated by the lack of new peaks.

Characteristic bands of the MPB shows phenolic O–H stretching at 327.29 cm^−1^ and ester carbonyl (C=O) at 1690.27 cm^−1^, while the peak of the aromatic ring vibrations is present as a broad peak from 1584.28 to approximately 1496.06 cm^−1^, with Ar–O–C ester stretching at 1262.17 cm^−1^ and C–O stretching of the ester at 1160.41 cm^−1^. All of the distinctive PMMA/PCL nanofibrous peaks are retained in the FTIR spectrum. Due to overlapping ester carbonyl groups, there is a little rise in intensity at 1723.46 cm^−1^. MPB ester groups are represented by additional weak bands at 1238.61 cm^−1^. There are no discernible peak shifts. MPB is successfully added to the nanofibres without changing the structure of the PMMA/PCL nanofibrous.

Similar bands to MPB are seen in PPB; however, the propyl chain is represented by extra aliphatic C–H extending around 2965.91–2885.15 cm^−1^. The peaks in the PPB-loaded PMMA/PCL nanofibres are the same as those in the blank PMMA/PCL. The ester carbonyl band showed a little increase. There are slight increases in the fingerprint area (1176.84–960.94 cm^−1^) and weak aliphatic C–H bands from the propyl group at approximately 2927.96 cm^−1^. There are neither any new peaks nor any characteristic peaks that disappear. PPB is inserted into the PMMA/PCL fibres with success and maintains its chemical stability.

### 3.3. Wettability of Nanofibrous Membranes

**Figure 3:**
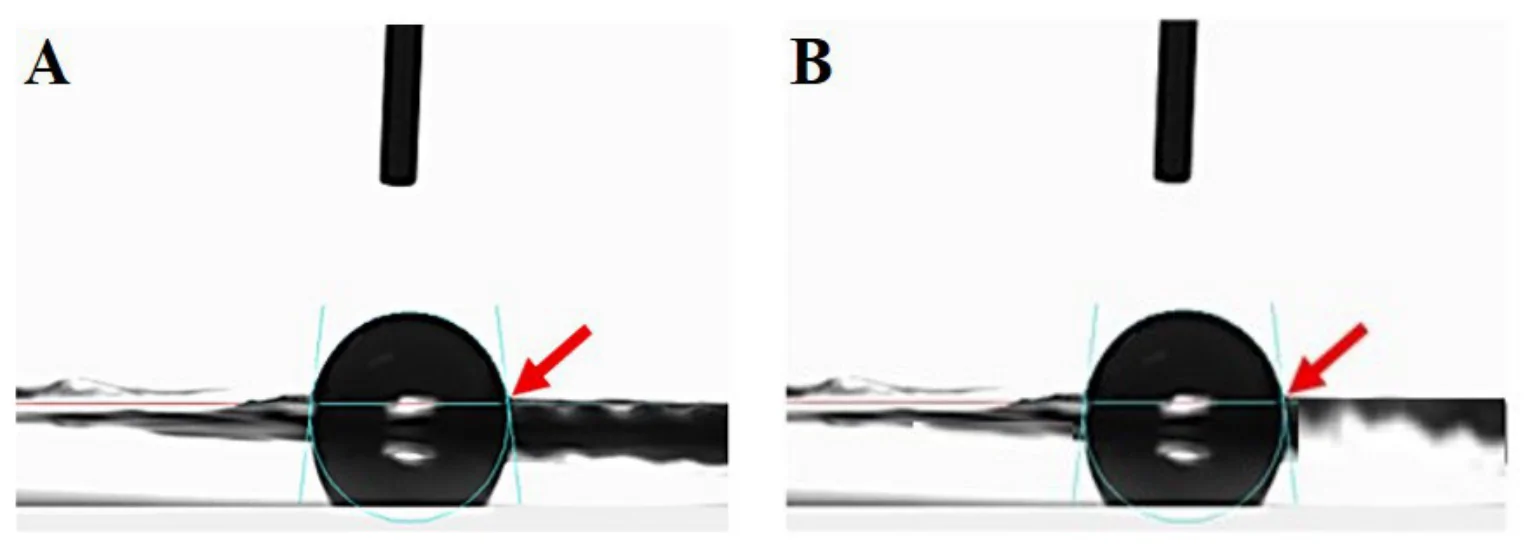
The contact angle for the PMMA/PCL nanofibrous membranes before (**A**) and after (**B**) loading with antiseptics. Red arrows represent the *θ* (<90°); blue lines represent the tangent to the wafer surface.

The wettability of the nanofibrous membranes was evaluated by contact angle measurements. The nanofibrous membranes exhibited a contact angle (*θ*) of 81.8 ± 1.8° (Figure 3), confirming their hydrophilic nature. The contact angles of the loaded PMMA/PCL nanofibrous membranes were similar to those of the pure PMMA/PCL nanofibrous membrane, suggesting no significant structural changes due to the addition of BA, MPB, and PPB in PMMA/PCL nanofibrous membranes (Figure 3).

### 3.4. XRD Analysis

**Figure 4:**
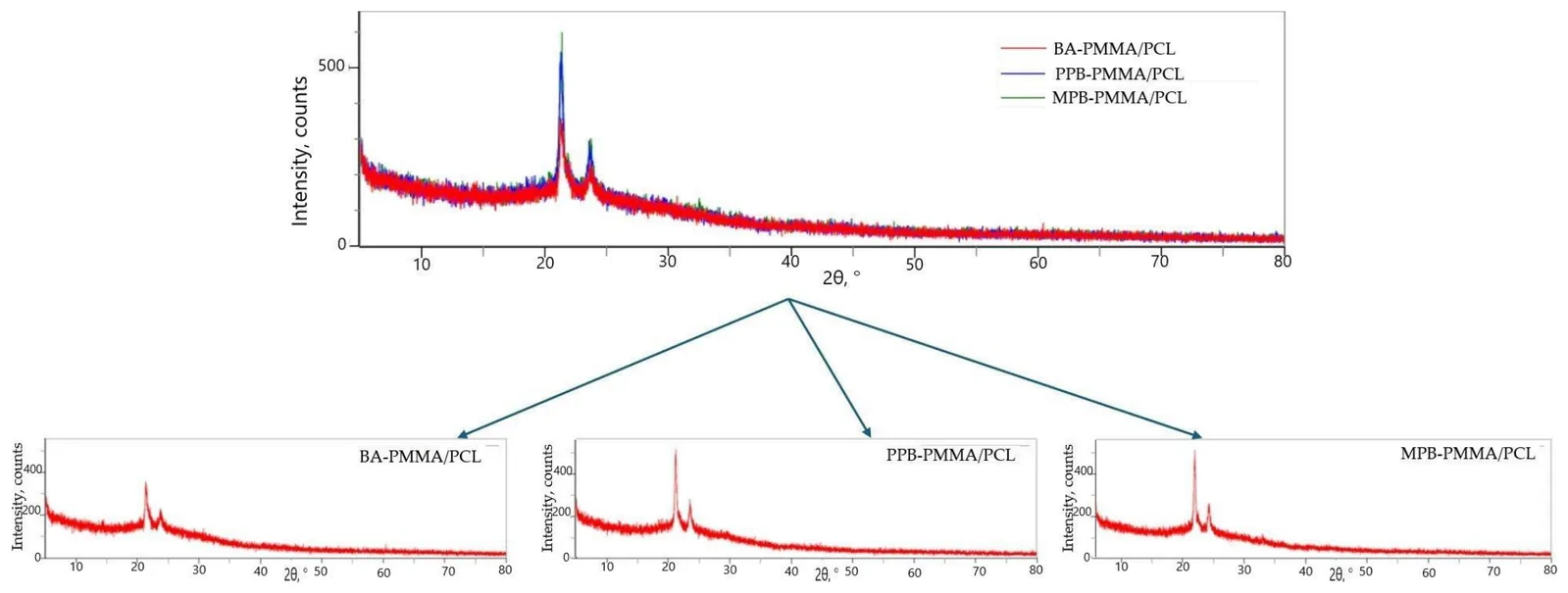
XRD patterns of antiseptic-loaded PMMA/PCL nanofibrous membrane. Arrows refer to the individual enlarged XRD patterns (**bottom**) separated from combined XRD patterns (**top**).

The crystallographic structure of electrospun nanofibrous membranes was obtained by XRD patterns as shown in Figure 4. XRD patterns of PMMA/PCL nanofibrous membranes reveal two broad patterns at 2θ 19.078° and 29.741° for PMMA and clear, intense patterns at 2θ 21.308° and 23.824° of PCL which indexed to amorphous and orthorhombic crystalline structure, respectively. The electrospinning technique and blending with PMMA appear to partially inhibit the crystallinity of PCL.

The diffractogram of the uploaded XRD pattern of the PMMA/PCL nanofiber coated with BA shows that the material is mostly amorphous with a few crystalline reflections that match BA. The large diffraction halo that extends from roughly 10° to 35° (2θ) in the XRD pattern of the PMMA/PCL membrane coated with BA indicates that the polymer matrix is primarily amorphous. While a weaker reflection near 23–24° may result from the overlapping crystalline planes of PCL and BA, a significant diffraction peak seen at about 21–22° shows the presence of crystalline BA. The lack of extra-sharp diffraction peaks indicates that benzoic acid is evenly distributed throughout the membrane without producing excessive crystalline domains and that the polymer matrix retains its amorphous structure after coating. The diffraction profile of the PMMA/PCL membrane coated with MPB or PPB is quite similar to the previous BA-coated sample based on the XRD pattern supplied, but it shows a higher peak intensity, indicating a higher degree of crystallinity of the loaded parabens. The typical crystalline reflections of semicrystalline PCL and the crystalline domains of the integrated parabens are represented by two separate diffraction peaks at roughly 21.5° and 23.8° (2θ). The successful deposition of the antimicrobial agent, while maintaining its partial crystallinity, is confirmed by the higher intensity of these peaks when compared to the unloaded membrane. The scanned region showed no additional diffraction peaks, indicating the lack of secondary phases or crystalline impurities. Overall, the findings show that the parabens stayed evenly distributed throughout the PMMA/PCL nanofibres, and that the coating procedure did not affect the PMMA/PCL nanofibres’ structural integrity.

### 3.5. Differential Scanning Calorimetry (DSC)

**Figure 5:**
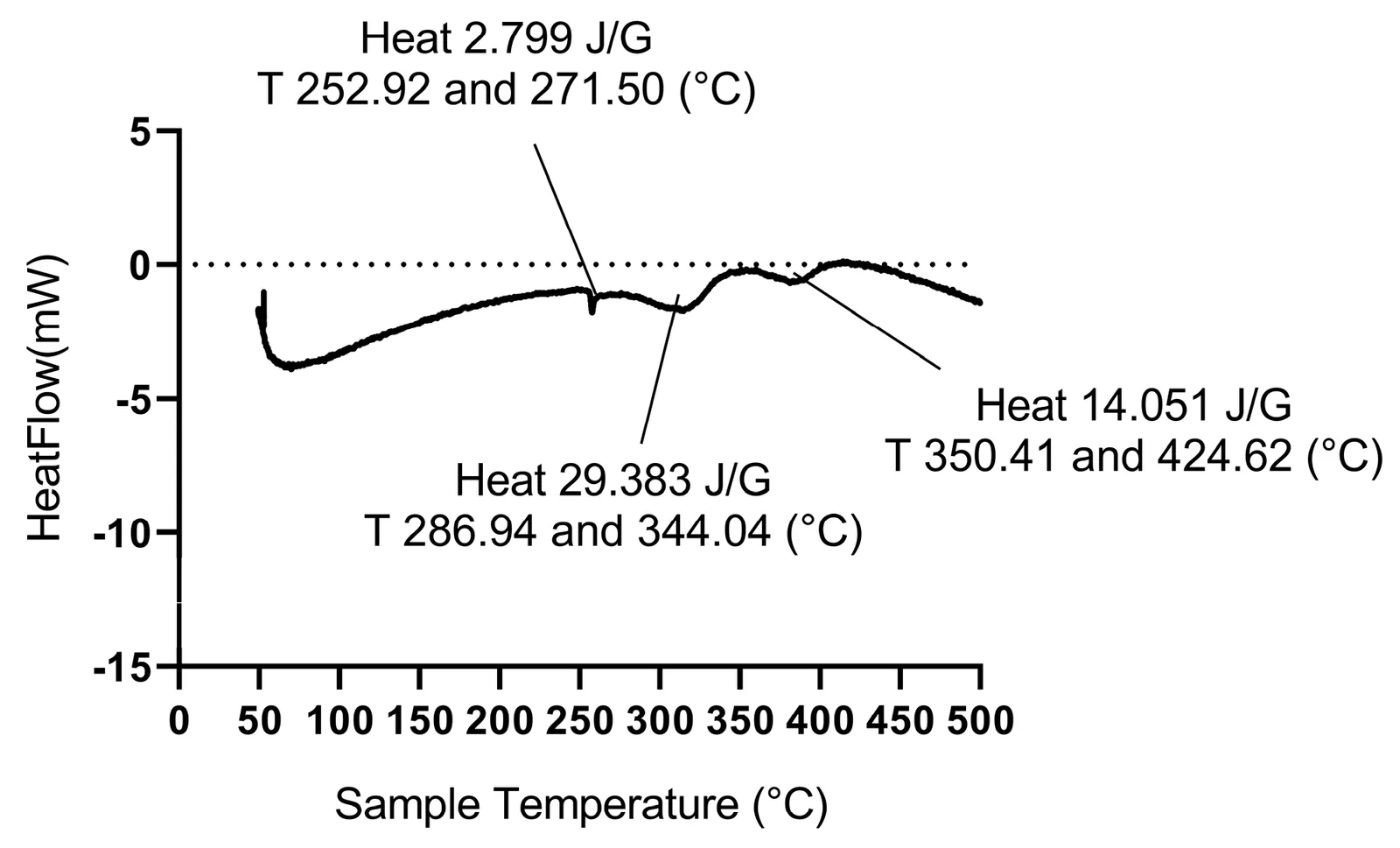
DSC graph of antiseptic-loaded PMMA/PCL nanofibrous membranes.

The thermal characteristics of the antiseptic-loaded nanofibrous membranes were examined using DSC analysis. The thermal behaviours of the antiseptic-coated PMMA/PCL nanofibrous membranes were similar to each other and also to that of the uncoated nanofibrous membranes; additionally, the *T_m_* and *ΔH_m_* of the nanofibrous membranes were insignificantly changed (*p* > 0.05). Figure 5 displays the primary thermal characteristics, such as the degree of crystallinity (X%), the enthalpy of melting (*ΔH_m_*), and the melting temperature (*T_m_*). Three different thermal events were visible in the DSC thermogram of the antiseptic-coated PMMA/PCL nanofibers. The thermal transition of surface-deposited antiseptics was identified as the cause of a minor endothermic peak at 252.92 and 271.50 °C (*ΔH* = 2.799 J g^−1^), suggesting the existence of a small quantity of free crystalline antiseptics on the nanofibre surface. The start of the nanofiber’s degradation is indicated by the principal endothermic event, which is centred at 286.94 and 344.04 °C (*ΔH* = 29.383 J g^−1^). This degradation mainly involves the breakdown of the PCL component with overlapping first PMMA degradation. PMMA depolymerisation is characterised by the appearance of a second degradation peak at 350.41 and 424.62 °C (*ΔH* = 14.051 J g^−1^). Effective solvent elimination during electrospinning is shown by the lack of notable thermal transitions below 200 °C. Overall, the DSC results show that the PMMA/PCL nanofibres’ thermal stability is unaffected by the antiseptic coating and that the composite retains the polymer blend’s distinctive two-step degradation behaviour.

### 3.6. Thermogravimetric Analysis (TGA)

**Figure 6:**
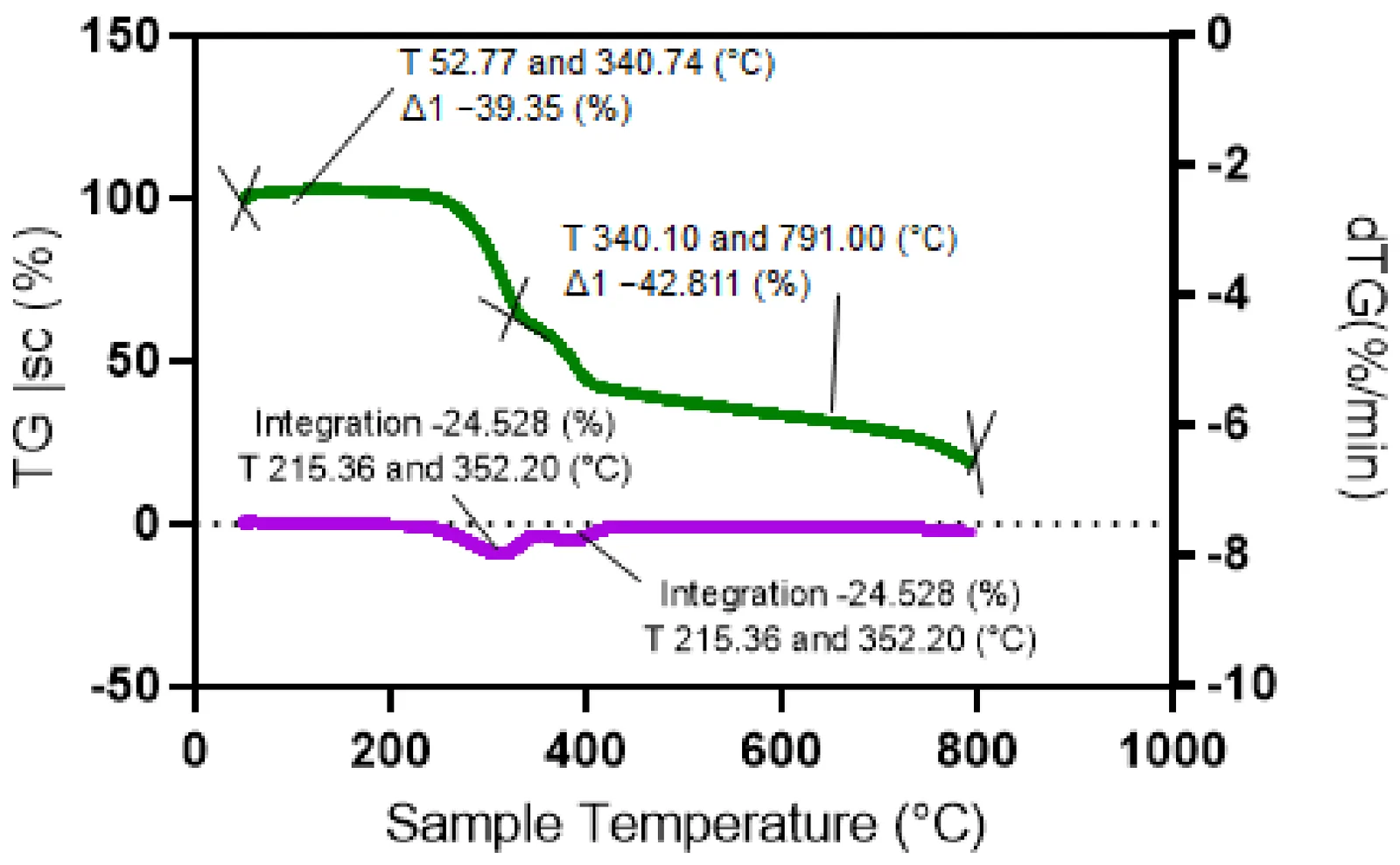
TGA graphs of antiseptic-loaded PMMA/PCL nanofibrous membranes.

Figure 6 displays the thermogravimetric analysis findings of the antiseptic-loaded PMMA/PCL nanofibrous membranes. The thermal behaviours of the antiseptic-coated PMMA/PCL nanofibrous membranes were similar to each other. Thus, the thermal stability of the coated PMMA/PCL nanofibrous membranes was not affected, implying that the antiseptics’ addition did not significantly alter (*p* > 0.05) the thermal stability of the PMMA/PCL nanofibrous membranes. The TGA/DTG thermogram of antiseptic-coated PMMA/PCL nanofibers shows the typical two-step thermal degradation behaviour of PMMA/PCL blends. The DTG curve (purple) shows the temperatures at which the rate of deterioration is greatest, whereas the TG curve (green) shows a steady loss of mass with rising temperature. The PMMA/PCL nanofibers coated with antiseptics showed a two-step degradation trend typical of polymer blends, according to the TGA study. With a maximal deterioration rate at 340.10 °C, the first stage of degradation took place between 52.77 and 340.74 °C, resulting in a 39.35% weight loss. This stage is linked to the antiseptic coating’s breakdown, the PCL phase’s deterioration, and the start of PMMA depolymerisation. The remaining PMMA matrix and carbonaceous intermediates broke down during the second degradation stage, which lasted from 340.10 to 791.00 °C and showed an additional 42.81% weight loss and a DTG maximum at 385.5 °C. At 791 °C, about 57.19% of the residual mass was still present (42.81% weight loss), suggesting that thermally stable char had formed. The second stage of degradation took place between 215.36 and 352.20 °C, resulting in a 24.53% weight loss. The second degradation from this stage lasted from 351.53 to 445.19 °C and showed an additional 6.414% weight loss.

### 3.7. Mechanical Stability of PMMA/PCL Nanofibrous Membranes

Tensile strength (MPa), Young’s modulus (MPa) and elongation at break of composite nanofibers were measured to study the mechanical stability of the membranes and to assess the effect of the antiseptics on the PMMA/PCL nanofibrous membrane, as displayed in Table 1. The uncoated membranes showed an average tensile strength of 13.48 ± 0.41 MPa, elongation at break of 17.60 ± 1.04% and Young’s modulus of 247.37 ± 9.53 MPa. No significant change (*p* > 0.05) was observed in tensile strength until the addition of PPB, but it significantly decreased (*p* ≤ 0.05) to 11.66 ± 1.10 MPa. Although the mean value for each of the experimental parameters under bending load was different between the coated and control samples, the difference was statistically significant (*p* ≤ 0.05) in elongation at break and Young’s modulus. The elongation at break gradually improved with the addition of antiseptics. The addition of antiseptics into the PMMA/PCL membrane significantly increased (*p* ≤ 0.05) its elongation value from 17.60 ± 1.04 to 19.48 ± 1.35, 18.85 ± 1.60 and 18.61 ± 1.24%, respectively. On the other hand, the addition of antiseptics into the PMMA/PCL membrane significantly increased (*p* ≤ 0.05) its Young’s modulus value from 247.37 ± 9.53 to 249.57 ± 6.48, 248.90 ± 7.25 and 248.12 ± 6.33 MPa, respectively. Interestingly, the incorporation of PCL into PMMA nanofibres significantly enhanced the mechanical strength. These results reveal that the compatibility between PMMA/PCL (70/30) could generate a porous, interconnected membrane that enhances the mechanical stability of the nanofibrous membrane.

### 3.8. Porosity, Hydrolytic Degradation and Swelling Behaviour (Water Intake) of Nanofibrous Membranes

**Figure 7:**
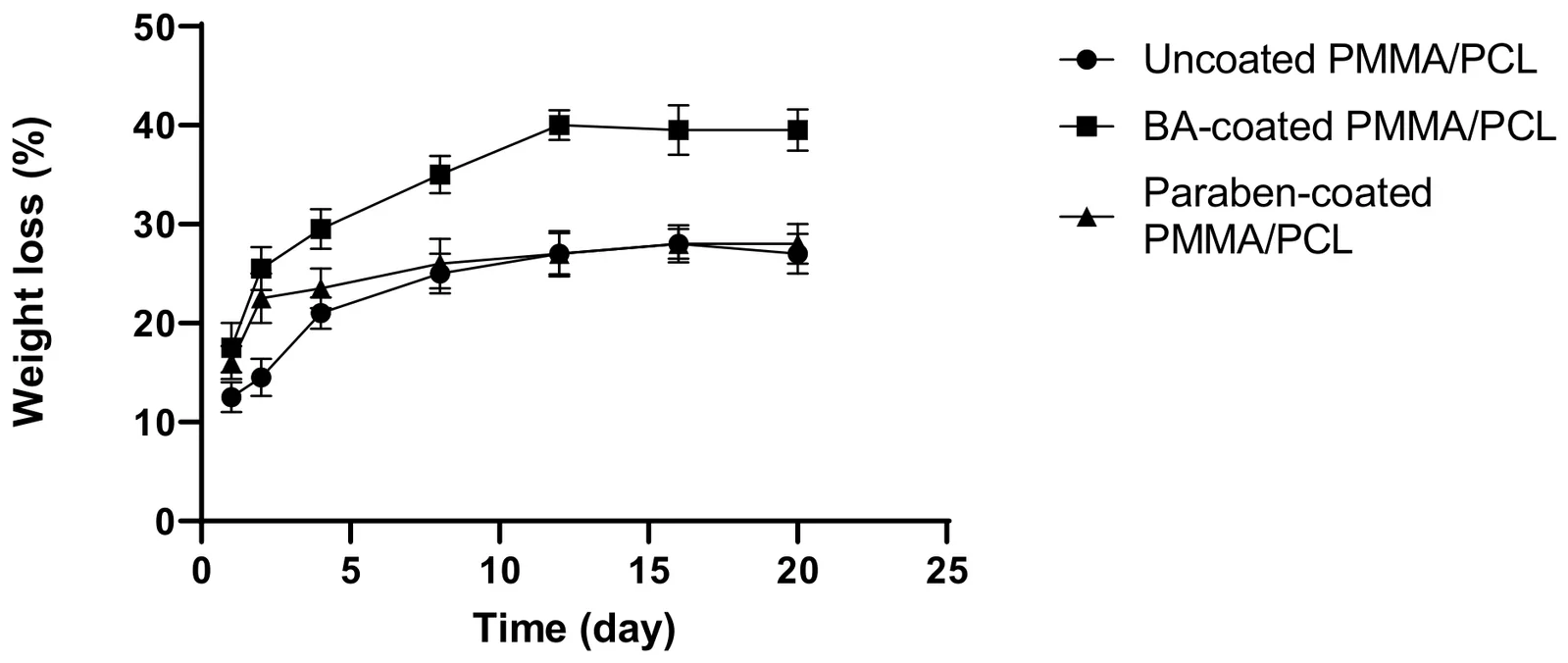
Weight loss (%) of the nanofibrous membranes.

**Figure 8:**
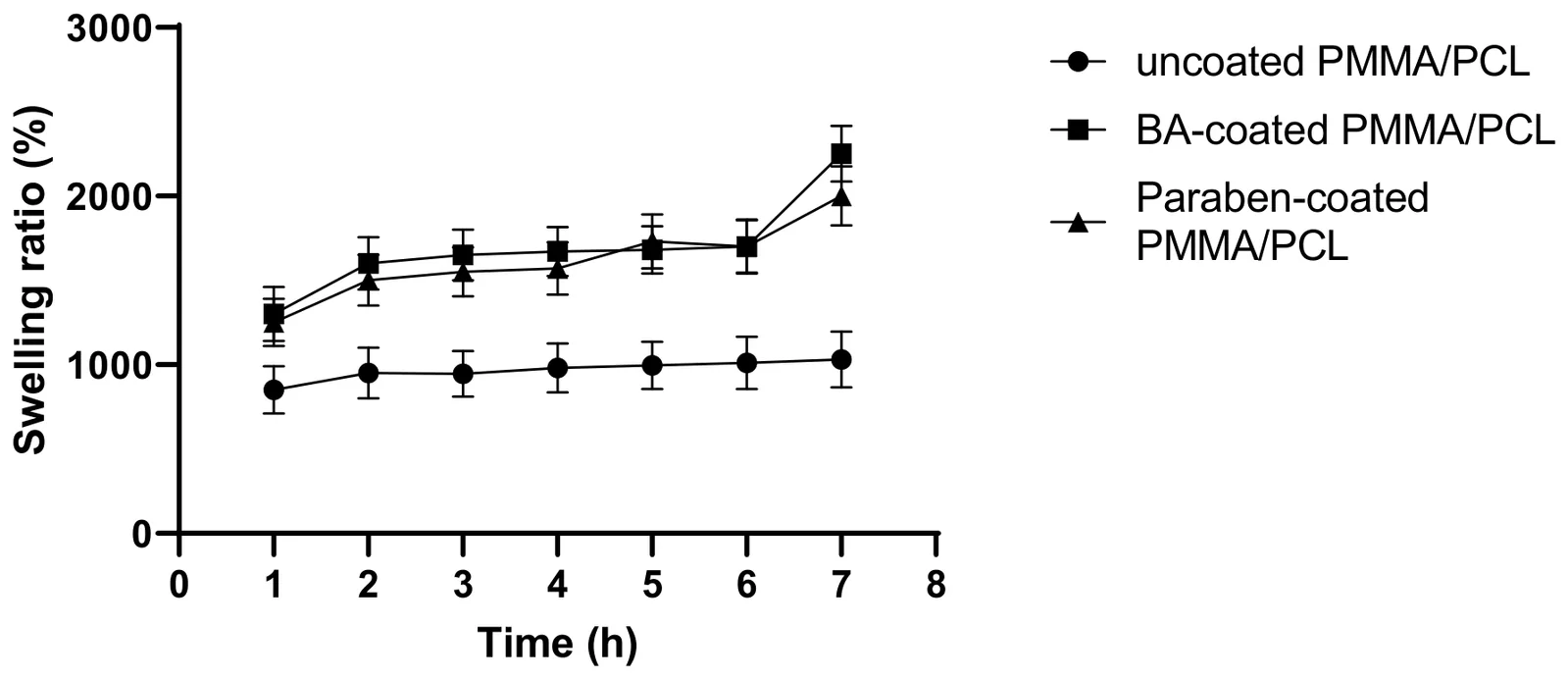
Swelling ratio (%) of the nanofibrous membranes.

The porosity of the nanofibrous membranes was determined before coating with antiseptics to ensure the efficiency of coating, and the findings revealed that the nanofibrous membranes had a porosity of 80 ± 0.55%. As shown in Figure 7 and Figure 8, nanofibrous membranes revealed different behaviour when submerged in SBF at pH 7.4, as a function of time. The swelling ratio of nanofibrous membranes was observed to be low and slow due to the high hydrophobicity of PMMA/PCL polymers and antiseptics as well. Therefore, coated nanofibrous membranes exhibit low weight loss behaviour in SBF (Figure 7), although coated nanofibrous membranes exhibit a high degree of degradation because they provide a high swelling ratio (Figure 8) compared to uncoated membranes. The nanofibrous membranes exhibited a slow weight loss during the first days, and then the sample mass tended to increase slowly in the following days.

### 3.9. BA, MPB and PPB Loading Efficiency and Capacity of PMMA/PCL Membranes

**Figure 9:**
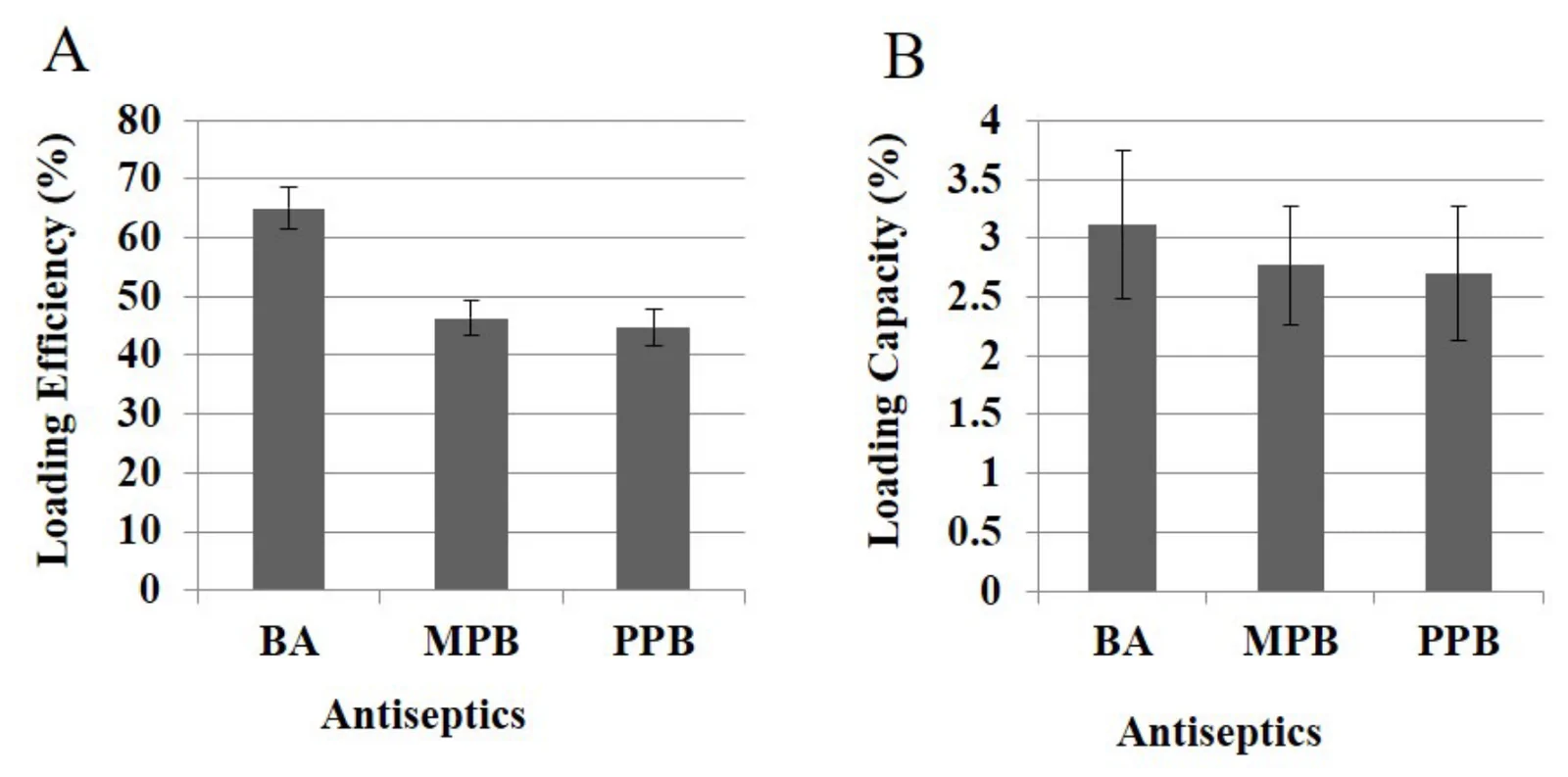
Loading efficiency (**A**) and loading capacity (**B**) of the PMMA/PCL scaffolds with benzoic acid (BA), methylparaben (MPB) and propylparaben (PPB).

The mean antiseptic loading efficiencies and capacities of the PMMA/PCL scaffolds with BA, MPB and PPB were estimated (Figure 9). All scaffolds showed loading efficiency of 65.07, 46.32 and 44.68% for BA, MPB, and PPB, respectively (Figure 9A). Their respective loading capacities were 3.11% for BA, 2.77% for MPB and 2.70% for PPB (Figure 9B).

### 3.10. The Sustained Release of BA, MPB and PPB-Loaded PMMA/PCL from Nanofibrous Membranes

**Figure 10:**
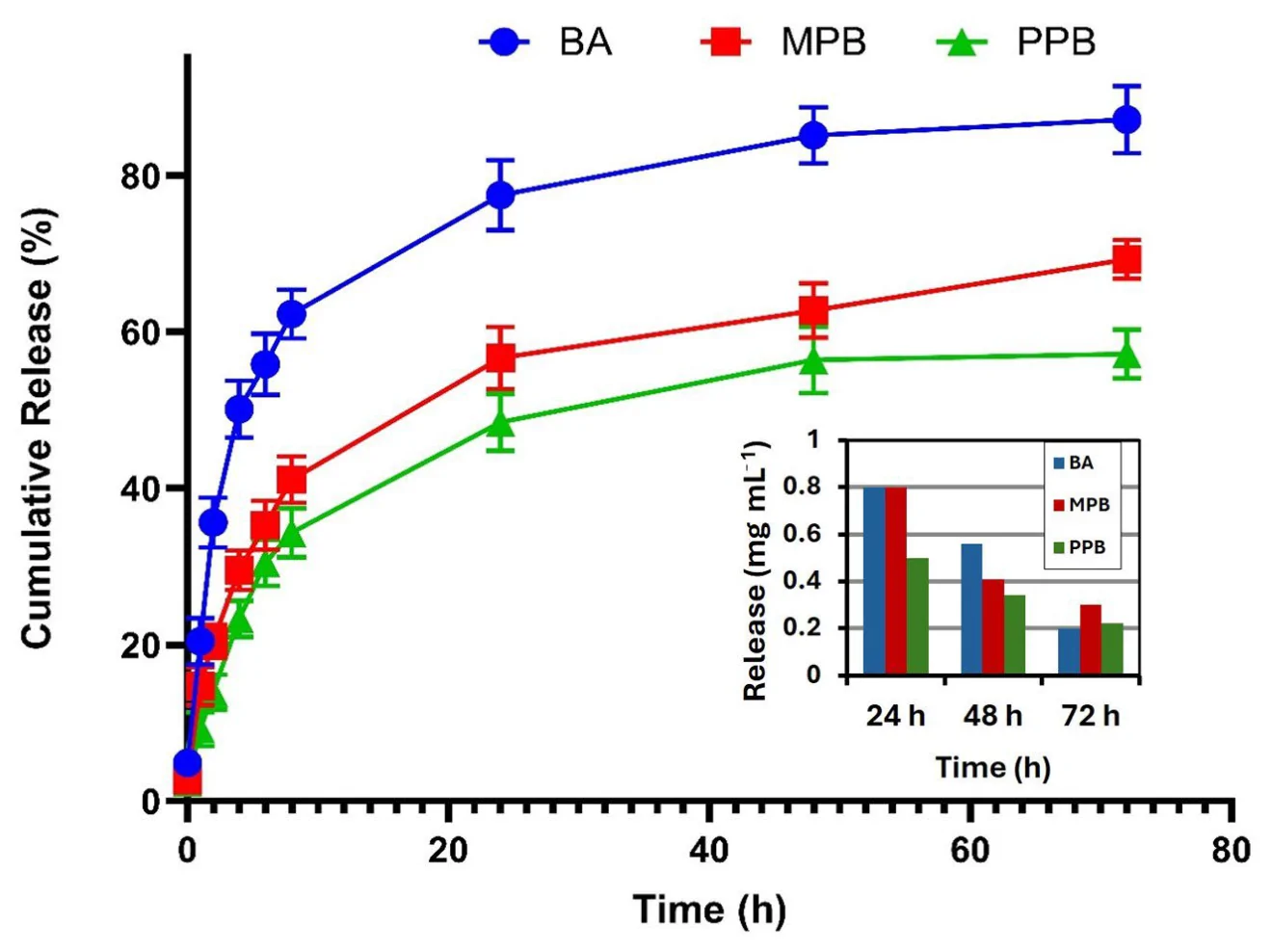
*In vitro* drug release profiles of BA-, MPB- and PPB-loaded nanofibrous membrane scaffolds.

The *in vitro* cumulative release profiles (%) of BA, MPB, and PPB over 72 h demonstrated a time-dependent increase in drug release for all formulations, with BA consistently exhibiting the highest release, followed by MPB and PPB. PMMA/PCL membranes (1 cm × 1 cm) loaded with the antiseptics were soaked in PBS and evaluated at different time intervals. The percentages of the *in vitro* cumulative release profiles of BA, MPB, and PPB were examined for 72 h. Data are expressed as mean ± SD. Statistical analysis using repeated two-way ANOVA followed by Tukey’s multiple comparisons test revealed no significant differences among the three groups at 0 h (*p* > 0.05). However, at 1 h, BA showed a significantly higher release compared to MPB (** *p* = 0.0057) and PPB (** *p* = 0.0021), while MPB was also significantly higher than PPB (**** *p* < 0.0001). At 2 h, BA remained significantly higher than PPB (*** *p* = 0.0002), whereas differences between BA and MPB, as well as MPB and PPB, were not statistically significant. From 4 h onward, a consistent and statistically significant trend was observed, where BA exhibited significantly higher releases than both MPB and PPB (**** *p* < 0.0001), and MPB showed significantly higher release than PPB (** *p* < 0.01 to **** *p* < 0.0001). At later time points (24, 48, and 72 h), these differences became more pronounced, confirming the superior release profile of BA compared to the other formulations (**** *p* < 0.0001). As shown in Figure 10, there was a time-dependent increase in drug release for all antiseptics. After 8 h, the burst release was 61.64 ± 4.11% for BA, 41.11 ± 2.98% for MPB and 34.32 ± 3.13% for PPB. BA consistently exhibited the highest release (87.15 ± 4.32%), followed by MPB (69.26 ± 2.46%) and PPB (57.18 ± 3.12) at the later time point of 72 h.

### 3.11. Cytotoxicity of Nanofibrous Membranes

**Figure 11:**
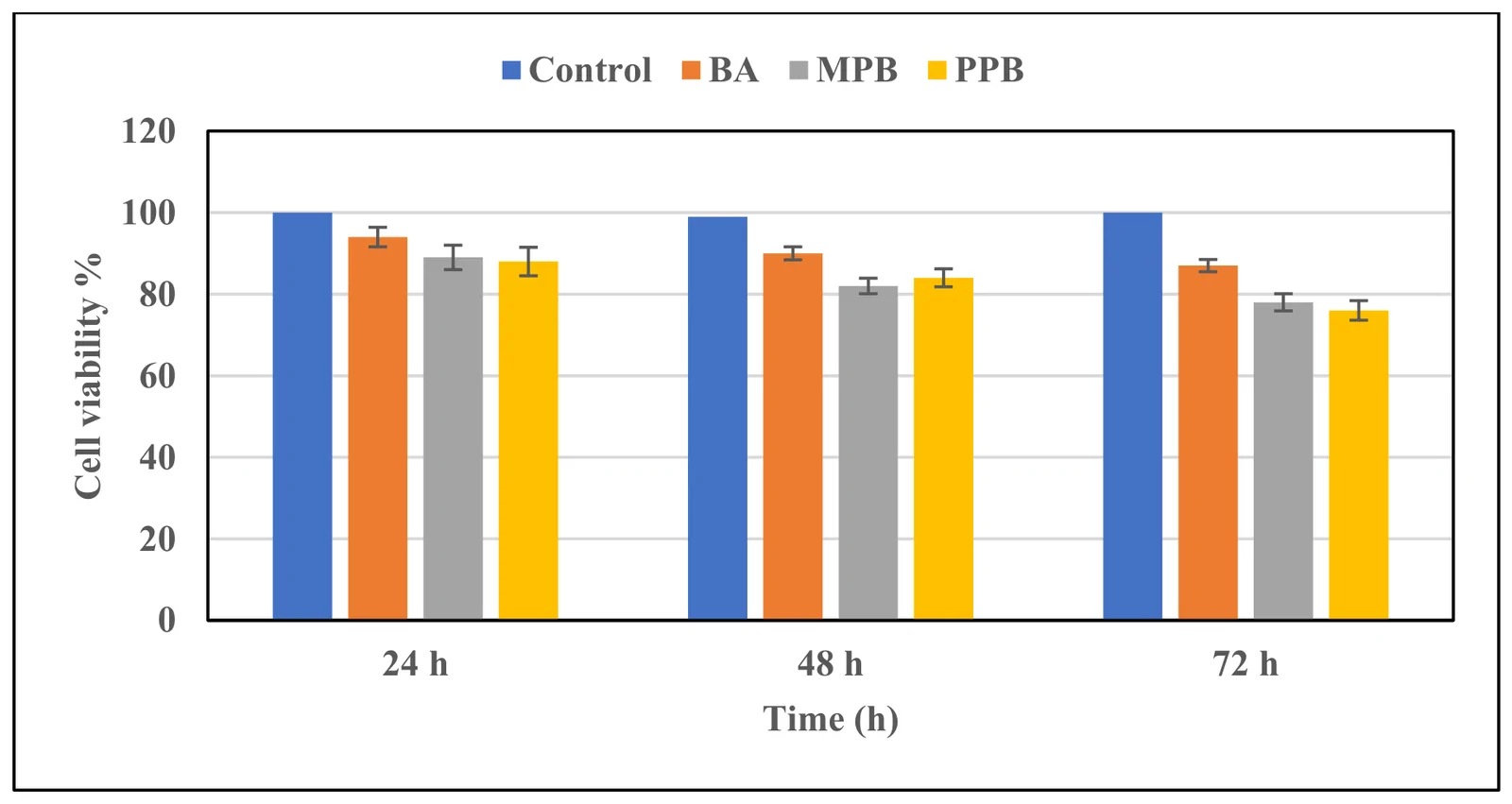
*In vitro* fibroblast cell viability after exposure to PCL/PMMA scaffolds loaded with BA, MPB and PPB for 24, 48 and 72 h.

The *in vitro* cytotoxicity of PCL/PMMA scaffolds loaded with BA, MPB and PPB was tested on a fibroblast cell line after 24, 48 and 72 h of exposure. According to Figure 11, the viability of fibroblast cells after 24 and 48 h exceeded 80% for all antiseptics. Even after 72 h, it exceeded 76% (Figure 11).

### 3.12. Resistance Profile of the Tested Isolates

**Figure 12:**
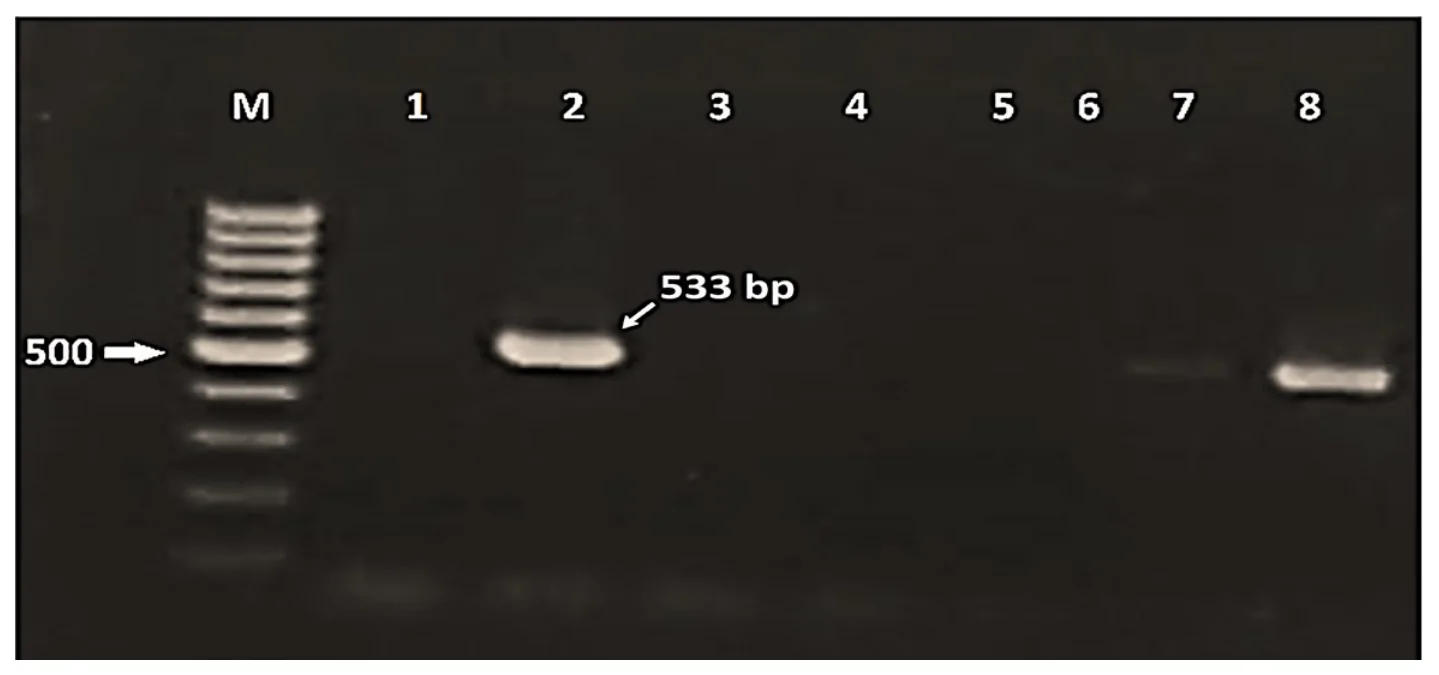
Amplicon size of the *mecA* gene in *Staphylococcus aureus* clinical isolates. M, 100 bp molecular size, lanes 2 and 8 are positive for *mecA*; lanes 1 and 3–7 are negative.

Cefoxitin-resistant *S. aureus* clinical isolates were screened for the presence of the *mecA* gene (Figure 12). *E. coli* clinical isolates were also tested for resistance against antibiotics from different classes, as summarised in Table 2. One MDR *E. coli* isolate, one MRSA isolate, and one fluconazole-resistant *C. albicans* isolate were selected for further experiments.

### 3.13. The Inhibitory Effect of Loaded PMMA/PCL Nanofibrous Membranes on Clinical Isolates

**Figure 13:**
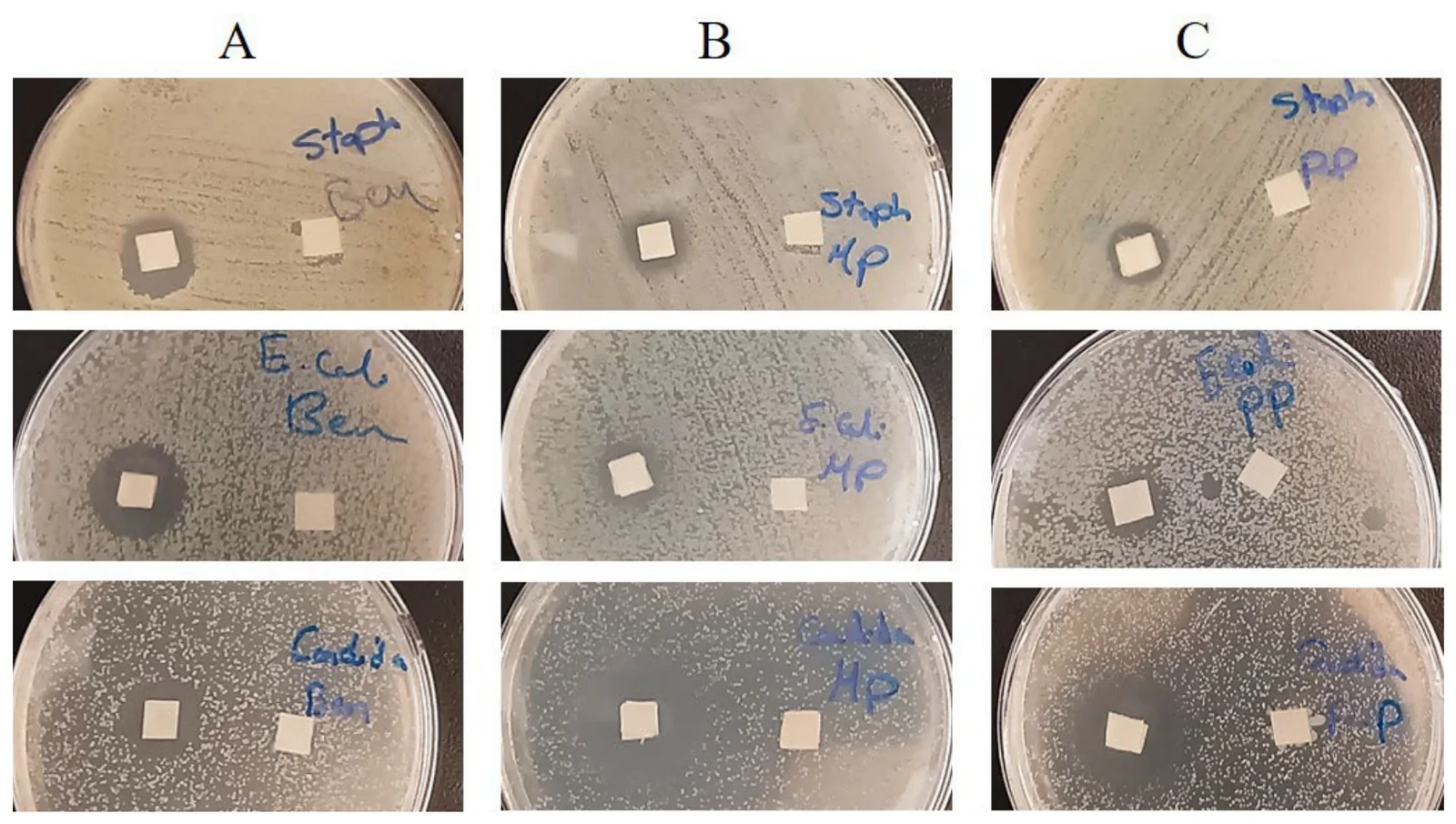
Inhibition zones produced by (**A**) BA-, (**B**) MPB- and (**C**) PPB-loaded PMMA/PCL nanofibrous membranes on the tested microorganisms.

The antimicrobial activity of 0.5 cm^2^ PMMA/PCL nanofibrous membranes loaded with BA, MPB, or PPB was evaluated against the tested isolates of *S. aureus*, *E. coli*, and *C. albicans*. While all the loaded nanofibrous membranes inhibited all tested microorganisms, the unloaded control pieces did not produce any zone of inhibition (Figure 13). Generally, the inhibitory effect of BA was more pronounced compared to MPB and PPB (Figure 13). The inhibition zones ranged between 14 and 20 mm in the case of bacteria and 19 and 20 mm in the case of *C. albicans* (Table 3).

### 3.14. Minimum Inhibitory Concentrations of Antiseptics on the Three Microorganisms

The MICs of the three antiseptics against the tested microorganisms ranged between 0.25 and 1 mg mL^−1^ (Table 4).

### 3.15. Inhibition of Microbial Biofilm Formation on PMMA/PCL Nanofibrous Scaffold Loaded with Antimicrobials

**Figure 14:**
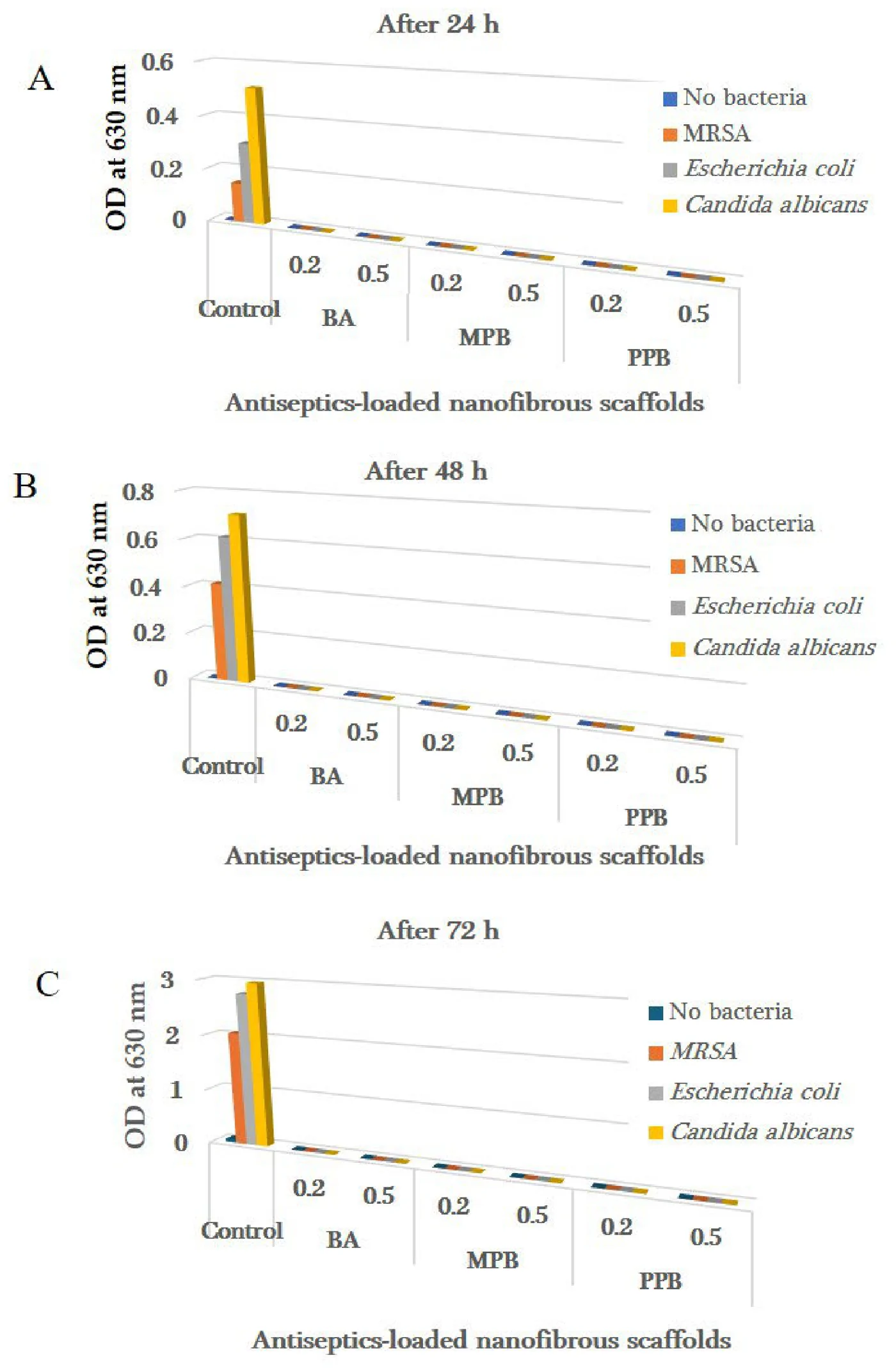
Quantification of biofilm formation by the tested microorganisms in the presence and absence of benzoic acid (BA), methyl paraben (MPB) and propyl paraben (PPB) after 24 h (**A**) 48 h (**B**) and 72 h (**C**).

The inhibition of biofilm formation by MRSA, *E. coli* and *C. albicans* was evaluated by the crystal violet method, viable counts of adhered microorganisms and scanning electron microscopy. As shown in Figure 14, the biofilm was completely inhibited by the antiseptics after 24, 48 and 72 h, as assessed by the crystal violet method. The inhibition of colonisation of the antiseptic-loaded PMMA/PCL membranes was also evaluated using the viable technique at 24 h, 48 h, and 72 h and is summarised in Table 5.

PMMA/PCL membranes loaded with all three antimicrobials completely inhibited biofilm formation by *C. albicans* on the nanofibrous membranes for up to 72 h (Figure 14). On the contrary, although no *S. aureus* and *E. coli* cells were detectable in the first 48 h, some bacterial cells were observed after 72 h on membranes loaded with the antiseptics. However, the microbial counts were significantly reduced by 6 to 7 logs (99.9999% reduction) compared with the control (Figure 14).

### 3.16. SEM Analysis of PMMA/PCL Nanofibrous Membrane Colonisation with Microorganisms

**Figure 15:**
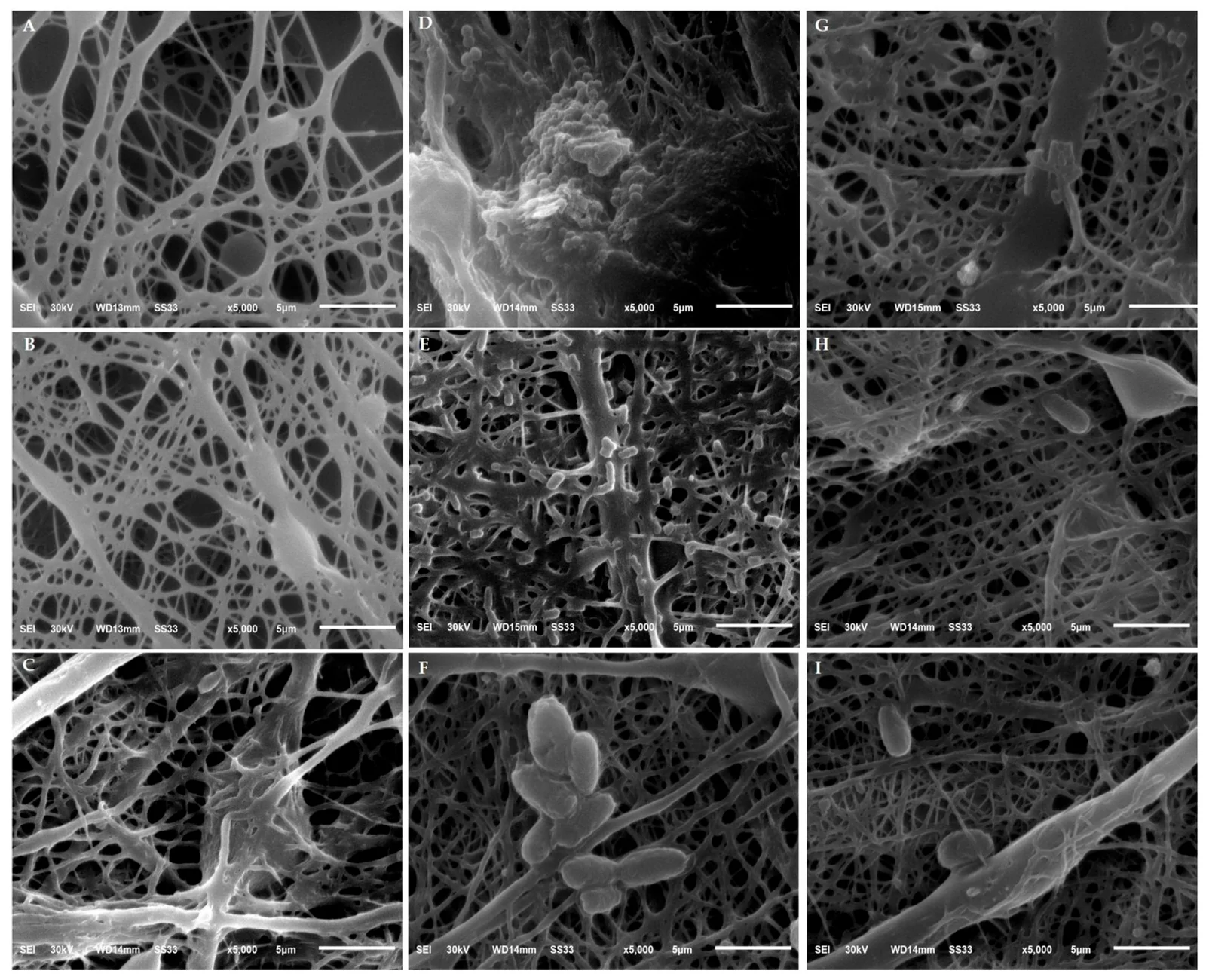
Scanning electron micrographs of bacterial adherence on the surface of PMMA/PCL nanofibrous scaffolds. Blank PMMA/PCL membranes (**A**–**C**), the bacterial colonisation of control PMMA/PCL membranes (**D**–**F**) and PMMA/PCL membranes loaded with BA after 72 h in nutrient broth inoculated with 10^5^ CFU mL^−1^ of MRSA (**D**,**G**), *Escherichia coli* (**E**,**H**), and *Candida albicans* (**F**,**I**).

The inhibitory effect of the PMMA/PCL membrane loaded with BA on the colonisation of the three investigated microorganisms was assessed by SEM (Figure 15). Figure 15A–C shows the pure PMMA/PCL membrane controls without bacteria (negative control). On the other hand, Figure 15D–F shows PMMA/PCL membranes with adhered bacteria (positive control). Figure 15G–I demonstrates the inhibition of biofilm formation on the BA-loaded PMMA/PCL membranes after 72 h. While the unloaded PMMA/PCL nanofibrous membranes were heavily colonised by MRSA (D), *Escherichia coli* (E) and *Candida albicans* (F), the BA-loaded PMMA/PCL membranes showed a few colonies of MRSA (G), *Escherichia coli* (H) and *Candida albicans* (I) that adhered to the nanofibrous membranes.

## 4. Discussion

The increasing rates of MDR pose public health risks. This led researchers to develop new microbial control strategies. This study employed a nanofibrous membrane scaffold for loading and sustaining the release of the broad-spectrum antimicrobials, BA, MPB, and PPB, for the inhibition of MDR microorganisms. The nanofibrous scaffolds were electrospun from equal parts PCL and PMMA. That blend has been commonly employed in diverse biomedical applications due to its biocompatibility, biodegradability, and mechanical properties [41]. In addition, the scaffold provides enough porosity to enhance haemostasis, tissue regeneration and healing and to protect the wound from microbial permeation [41]. Although the porosity and surface area of the nanofibrous mat would increase by increasing the amount of PMMA, that was avoided for fear of the increase of beads in the scaffold and the decrease in its degradability [26].

Electron microscopy revealed that the electrospinning of a blend of equal ratios of PMMA and PCL generated a 3D mat with high porosity and large surface area that helps to enhance the adhesion of cells and the loading and delivery of drugs [42].

ATR-FTIR spectroscopy analysis revealed full physical incorporation of the PMMA/PCL blend in the nanofibrous scaffold. That was demonstrated by the presence of the specific absorption bands of PCL related to C=O, CH_2_ bending, and C–O–C modes at 1722.12, 1365.35, and 1178.29 cm^−1^, respectively [43]. In addition, there were characteristic absorption peaks of PMMA at 1722.12 and 1240 cm^−1^, assigned to the stretching vibration of C=O bonds and the stretching vibration of the O–CH_3_ of the ester group, respectively [30]. Signals observed at 1140 and 900 cm^−1^ reflected the vibrations of C–C and C–H bonds of methyl groups. The double band was observed at 2952 and 2995 cm^−1^, suggesting the characteristic stretching vibrations of the C–H bonds of the methyl group [44]. The PMMA/PCL blend spectrum exhibited a double split band related to the C–H stretching band that appeared at 2942.84 and 2865.7 cm^−1^ and the C–H stretching band that appeared at about 3000 cm^−1^ [45]. On the other hand, new peaks appeared at 1202.12 cm^−1^, indicating a compatibility between PMMA and PCL [45].

The PMMA/PCL nanofibrous membrane was successfully fabricated and loaded with BA, MPB, and PPB, according to the FTIR spectra. There was no covalent chemical reaction between the antiseptics and the PMMA/PCL nanofibrous membrane, as evidenced by the preservation of the distinctive functional groups of both the polymers and the antimicrobial agents as well as the absence of new absorption bands or significant shifts in peak positions. Nevertheless, slight variations in peak broadening and intensity, especially at 1722.12 cm^−1^, indicate weak intermolecular interactions including van der Waals forces and hydrogen bonds, which are anticipated following drug encapsulation.

The XRD patterns of PMMA/PCL nanofibers show two broad patterns at 2θ 13° and 29° for PMMA and distinct intense patterns at 2θ 21.6° and 24° for PCL [25]. These patterns correspond to orthorhombic and amorphous crystalline structures, respectively [45]. Additionally, XRD patterns of PCL/PMMA blend membranes revealed decreased drug and PCL crystallinity, which was a crucial sign of drug and polymer miscibility [45]. In the past, the rate of solvent evaporation had an impact on the degree of crystallinity [46]. This suggests that the PCL content in the membrane may increase the crystallinity of drug-loaded PMMA/PCL. These findings are in line with those of Elashmawi et al. [47], who discovered that adding PMMA to PVDF reduced the crystallinity structure and made the two polymers totally miscible. According to Saleh et al. [48], the nanofibrous membranes are formed like a matrix. This work aims to investigate the electrospinning of PMMA and PCL. Compared to pure PMMA and PCL, the blended fibres used to make these spun-woven mats are significantly finer. X-rays were used to measure the degree of crystallinity (X%). Furthermore, PMMA had two diffraction peaks at 2θ = 21.4° and 23.8° that are attributed to (110) and (200), which highlight the crystal planes of semi-crystalline PCL, in addition to massive amorphous humps at 2θ = 15°. Effective interaction and mixing between the two may be shown by variations in the intensity and location of particular absorption bands seen in FT-IR spectra.

The contact angle measurement determined the wettability of the nanofibrous membrane. This parameter is important as it reflects the hydrophobic/hydrophilic nature of nanofibrous membranes [49]. Materials are classified as hydrophilic if the contact angle is less than 90° and hydrophobic if the contact angle values range from 90 to 120° [44]. In this study, the prepared nanofibrous scaffold showed a contact angle value of 81.8 ± 1.8°, which confirms the hydrophilic nature of the scaffold. The hydrophilicity of a wound-healing scaffold is usually important and vital to enable the material to absorb wound exudate, facilitate nutrient and gas exchange, and maintain a moist environment [50].

The combined DSC and TGA analyses show that the thermal stability of the polymer blend is not considerably compromised by coating the PMMA/PCL nanofibers with benzoic acid. Rather, the material maintains its distinctive two-step deterioration profile, with the first significant breakdown taking place around 310 °C and the second around 385 °C, leaving a significant residual char (~18%), which is a sign of strong thermal resistance. These findings confirm that the coated nanofibers are appropriate for uses requiring sterilizing or mild heat exposure without premature deterioration. The two-stage degradation mechanism was confirmed by the DTG peak temperatures, which closely matched the endothermic transitions seen in the DSC thermogram. The coated PMMA/PCL nanofibers showed good thermal stability overall, with deterioration taking place well above processing and biomedical application temperatures.

A common method for measuring the tensile strength of thin scaffolds was applied for the analysis of the mechanical properties [51]. Salim et al. [25], revealed that the incorporation of low concentration of PCL (2, 4%, *w*/*v*) into PMMA nanofibres significantly enhanced the mechanical strength, compared to nanofibers with high concentration of PCL (8%) and scaffolds without PCL, leading to the compatibility between PMMA and PCL as a blending mechanism to incorporate the drugs in the mixture and also the nanofibers. Meanwhile, the incorporation of 1% mercaptopurine (6-MP) to sandwich the form of PCL and PMMA solution could generate porous interconnected scaffold that enhances mechanical stability of nanofibrous mats and in turn facilitates cell proliferation, adhesion and differentiation. The stress–strain curves for samples can be characterised as an initially elastic response, followed by a short inelastic region and then a sudden descending response due to failure of the specimen.

The general mechanical properties of the pure PCL film are known to be characterised by extremely high elongation at break and average tensile strength of 20–30 MPa [52]. As in Table 1, the uncoated membranes showed an average tensile strength of 13.48 ± 0.41 MPa, elongation at break of 17.60 ± 1.04% and Young’s modulus of 247.37 ± 9.53 MPa. No significant change (*p* > 0.05) was observed in tensile strength until the addition of PPB, but then it significantly decreased (*p* ≤ 0.05) to 11.99 ± 1.10 MPa. This decrease might be due to the increased level of antiseptic contents in the PCL matrix, because it is known that typical additives except for cross-linking agents decrease tensile strength and increase elongation of the film [53]. Significant change (*p* ≤ 0.05) was observed in elongation at break and Young’s modulus between loaded and unloaded membranes.

Although high porosity is desirable from the biological point of view, this must be balanced with the corresponding reduction in mechanical properties so that the structural integrity of the biomaterial is not compromised [54]. The high surface area to volume ratio, the high porosity, the biocompatibility, mechanical strength, and degradability, which make electrospun fibres of polymers good candidates for soft tissue engineering, are the criteria of the natural extra cellular matrix [55,56].

Coated nanofibrous membranes absorb water or physiological fluids through their internal pores, this can assist cell signalling and nutrition in membranes or provide route for the egress of drugs [57]. As shown in Figure 7 and Figure 8, nanofibrous membranes revealed different behaviour when submerged in SBF at pH 7.4, as a function of time. Coated nanofibrous membranes exhibited a high degree of degradation because they provided a high swelling ratio compared to uncoated membranes. This can be ascribed to the miscibility and amorphous structure of nanofibrous membranes as aforementioned and proven by XRD analysis, which thus allows more water molecules to diffuse through pores and swell the membranes even further.

The PMMA/PCL nanofibrous scaffold was loaded with one of three broad-spectrum antimicrobials, namely, BA, MPB, and PPB. Those antimicrobials were chosen because the FDA categorises them as safe antimicrobials [58]. They have been widely used as preservatives for over 70 years in the food, drug, and cosmetic industries due to their antibacterial and antifungal properties [59]. In addition, BA is also used topically for the treatment of fungal skin infections [60]. We utilised the poor water-solubility of BA, MPB, and PPB [9] and dissolved them in alcohol/acetone. The PMMA/PCL nanofibrous membrane scaffolds were impregnated in these solutions, drained, and air-dried, to leave behind a layer of the antimicrobials on the large surface area of the nanofibrous scaffolds. In this way, the loaded antimicrobials on the surface of the nanofibrous membranes would act as reservoirs for the antimicrobials and would release them slowly into the wound aqueous environment.

Neither the nanofibrous scaffold nor the antiseptic-loaded nanofibrous scaffolds affected the viability of the fibroblast cell line for 72 h, suggesting their non-cytotoxicity. The safety of the PCL/PMMA nanofibrous membranes was previously reported [61], and therefore, the PCL/PMMA membranes have been considered a safe scaffold for cell proliferation [26]. The percentages of fibroblast cell survival in the presence of PMMA/PCL nanofibrous membrane scaffold loaded with the tested antiseptics exceeded 80%. After 72 h, the survival was more than 76%, which, according to ISO 10993-5, is considered non-cytotoxic [62,63]. However, it should be mentioned that although BA, MPB, and PPB are categorised as safe antimicrobials and approved by the FDA [27] as preservatives in pharmaceuticals and cosmetics, there are some concerns that they may exhibit a dose-dependent cytotoxicity, like mitotic toxicity, chromosomal aberrations, and DNA damage, especially at high concentrations [64,65,66,67]. Nonetheless, the general consensus of both the scientific community and regulatory agencies indicates that the three antimicrobials could be safely used at typical recommended concentrations without harmful effects on human health [67].

The antimicrobial activity of the PMMA/PCL nanofibrous membrane scaffolds loaded with antimicrobials was challenged with three selected MDR microorganisms, namely, MRSA (Gram-positive), *E. coli* (Gram-negative) and *C. albicans* (fungus). MRSA is a commensal that colonises the human skin, nasal nares, mouth and gastrointestinal tract [35]. It readily acquires antimicrobial resistance through mutation or horizontal transfer of resistance genes [68]. *E. coli* inhabits the intestinal tract and is considered one of the most frequent causes of infections, including wound infections. It serves as a reservoir for acquiring and disseminating antimicrobial resistance genes [44]. On the other hand, *C. albicans* is a normal commensal that acts as an opportunistic pathogen, and its presence in surgical wounds and occlusive dressings delays their healing [69].

Unlike antibiotics, BA, MPB, and PPB have no specific target. They inhibit microbial growth by interfering with metabolic functions, disrupting cell membrane integrity, altering pH balance, and inhibiting protein, DNA and RNA synthesis in bacterial and fungal cells [70]. As a result, despite their widespread and extensive use in food, cosmetics, and medicines, there is no evidence of acquired resistance by mutation, efflux, or plasmid-mediated mechanisms, and resistance has not been reported [71].

Generally, BA and parabens are used as preservatives at concentrations ranging between 0.5 and 1 mg/mL and 0.2 and 3 mg/mL, respectively [69,72]. The PMMA/PCL nanofibrous membrane scaffold loaded with BA, MPB, or PPB continued to release the antimicrobials for three days at concentrations inhibitory to the tested microorganisms. That was demonstrated by agar diffusion and the ability of the drugs to inhibit biofilm formation on all the antiseptic-loaded nanofibrous membranes by the tested MDR organisms.

The cumulative release suggested a time-dependent increase in the release of all antiseptics. The loading efficiency of BA, MPB, and PPB was 65.07, 46.32 and 44.68%, respectively. BA consistently exhibited a significantly higher release over the 72 h (*p* = 0.0057 to 0.0001), followed by MPB and PPB. That suggests the superior release profile of BA compared to the other formulations. The initial release of all the antiseptics was high and then sustained, as previously reported [73]. That is probably because of the dissolution of the loosely deposited antiseptics on the surface of the nanofibrous membranes, which rapidly dissolve upon contact with the releasing medium [73].

In this study, the MICs of the three antiseptics against *S. aureus*, *E. coli* and *C. albicans* ranged from 0.25 to 1.0 mg mL^−1^. Therefore, the released antiseptics from the PMMA/PCL nanofibrous membrane scaffold, which ranged between 0.20 and 0.8 mg mL^−1^ during the 72 h, were inhibitory to the tested organisms. That was demonstrated by the crystal violet technique, viable count and electron microscopy. The crystal violet technique was the least accurate and failed to detect the low count of microorganisms, which were detected by both the viable count technique and electron microscopy. The viable count demonstrated that the microorganisms were reduced to non-detectable levels or significantly reduced the counts of colonising for the tested microorganisms by 6 to 7 logs (99.9999% reduction), compared with the control, for 72 h.

## 5. Conclusions

The PMMA/PCL nanofibrous scaffold, with its increased surface-to-volume ratio, loaded with the poorly soluble, broad-spectrum antimicrobials BA, MPB, and PPB, successfully sustained their release for 3 days at inhibitory levels against MDR microorganisms. Because the scaffolds loaded with antiseptics are non-cytotoxic and have broad-spectrum activity against MDR microorganisms, they could be recommended for use as wound dressings, particularly in non-healing wounds infected with MDR microorganisms. This would be particularly useful in diabetic patients, pressure ulcers, and other non-healing surgical wounds. The strategy could also be utilised to coat the surface of dentures with a scaffold loaded with antimicrobials to reduce their microbial colonisation and mitigate problems like stomatitis.

## Figures and Tables

**Table 1:** Mechanical properties of the coated and uncoated PMMA/PCL nanofibrous membranes.

Nanofibrous Membranes	Tensile Strength (MPa)	Elongation at Break (%)	Young’s Modulus (MPa)
PMMA/PCL	13.48 ± 0.41 ^a^	17.60 ± 1.04 ^a^	247.37 ± 9.53 ^a^
BA-coated PMMA/PCL	12.60 ± 1.09 ^ab^	19.48 ± 1.35 ^b^	249.57 ± 6.48 ^b^
MPB-coated PMMA/PCL	12.11 ± 0.86 ^ab^	18.85 ± 1.60 ^b^	248.90 ± 7.25 ^b^
PPB-coated PMMA/PCL	11.99 ± 1.10 ^c^	18.61 ± 1.24 ^b^	248.12 ± 6.33 ^b^

Data are expressed as the mean ± SD (*n* = 3). The different uppercase letters in the same column indicate a significant difference (*p* ≤ 0.05), as assessed by Duncan’s multiple range test.

**Table 2:** Resistance profile of the tested microorganisms.

No	Microorganism	Antibiotic Resistance Pattern
1	MRSA	OXA, CEF, ERE, CIP, GEN, TET, SXT, FUS
2	*E. coli*	CTX, CRO, CIP, SXT, GEN, ATM, TET
3	*C. albicans*	FLU

**Table 3:** Inhibition zones produced by antiseptic-loaded scaffolds against *Staphylococcus aureus*, *Escherichia coli* and *Candida albicans*.

Microorganism		Antiseptic-Loaded Scaffolds *		
	Control	BA	MPB	PPB
		Inhibition zone diameter in mm		
*S. aureus*	0	17 ± 0.5	15 ± 0.8	14 ± 0.3
*E. coli*	0	20 ± 1.0	15 ± 0.5	15 ± 0.5
*C. albicans*	0	19 ± 1.0	20 ± 1.0	20 ± 0.8

* BA, benzoic acid; MPB, methylparaben; PPB, propylparaben.

**Table 4:** Minimum inhibitory concentrations of benzoic acid, methylparaben, and propylparaben (mg mL^−1^) on three microorganisms.

Microorganism	BA	MPB	PPB
MRSA	0.5	1.0	0.25
*Escherichia coli*	0.25	0.5	0.5
*Candida albicans*	0.25	0.25	0.25

**Table 5:** Inhibitory effects of benzoic acid, methylparaben and propylparaben on the colonisation of MRSA, *Escherichia coli* and *Candida albicans* to the surface of loaded PMMA/PCL membrane at 24, 48 and 72 h.

Antimicrobial	MRSA			*Escherichia coli*			*Candida albicans*		
	Time in Hour								
	24	48	72	24	48	72	24	48	72
	Bacterial Counts (CFU * mL^−1^)								
Control	5.0 × 10^6^ ± 2.0 × 10^2^	2.0 × 10^8^ ± 2.0 × 10^6^	1.0 × 10^9^ ± 1.0 × 10^2^	5.0 × 10^6^ ± 1 × 10^5^	2.0 × 10^8^ ± 1 × 10^6^	1.0 × 10^9^ ± 1.0 × 10^2^	5.0 × 10^6^ ± 1.0 × 10^4^	2.0 × 10^8^ ± 1.0 × 10^5^	1.0 × 10^9^ ± 1.0 × 10^6^
Benzoic acid	ND ^♦^	ND	2.2 × 10^2^ ± 7.0 × 10	ND	ND	1.2 × 10^2^ ± 5.0 × 10	ND	ND	ND
Methylparaben	ND	ND	ND	ND	ND	1.6 × 10^2^ ± 4.0 × 10	ND	ND	ND
Propylparaben	ND	ND	ND	ND	ND	2.6 × 10^2^ ± 6.0 × 10	ND	ND	ND

* CFU, colony forming units; ^♦^ ND, not detected.

## Data Availability

The corresponding author can provide the data used in this study upon reasonable request.

## Abbreviations

The following abbreviations are used in this manuscript:

BA	benzoic acid
*E. coli*	*Escherichia coli*
*C. albicans*	*Candida albicans*
DMEM	Dulbecco’s Modified Eagle Medium
FTIR	Fourier transform infrared spectroscopy
HSF	Human skin fibroblast
MDR	multiple drug resistant
MPB	Methylparaben
MHA	Muller-Hinton agar
MHB	Muller-Hinton broth
MRSA	methicillin resistant *Staphylococcus aureus*
PCL	Polycaprolactone
PMMA	polymethyl methacrylate
PCL	poly (ε-caprolactone)
PPB	propylparaben
SEM	scanning electron microscope
*S. aureus*	*Staphylococcus aureus*
TSB	Tryptic soya broth

## References

[1] Fongang H., Mbaveng A.T., Kuete V. (2023). Global burden of bacterial infections and drug resistance. Advances in Botanical Research.

[2] Jernigan J.A., Hatfield K.M., Wolford H., Nelson R.E., Olubajo B., Reddy S.C., McCarthy N., Paul P., McDonald L.C., Kallen A. (2020). Multidrug-resistant bacterial infections in US hospitalized patients, 2012–2017. N. Engl. J. Med..

[3] Dadgostar P. (2019). Antimicrobial resistance: Implications and costs. Infect. Drug Resist..

[4] Yoneyama H., Katsumata R. (2006). Antibiotic resistance in bacteria and its future for novel antibiotic development. Biosci. Biotechnol. Biochem..

[5] Luepke K.H., Mohr J.F. (2017). The antibiotic pipeline: Reviving research and development and speeding drugs to market. Expert Rev. Anti-Infect. Ther..

[6] Gajic I., Tomic N., Lukovic B., Jovicevic M., Kekic D., Petrovic M., Jankovic M., Trudic A., Culafic D.M., Milenkovic M. (2025). A comprehensive overview of antibacterial agents for combating Multidrug-Resistant bacteria: The current landscape, development, future opportunities, and challenges. Antibiotics.

[7] Yu Q., Wu Z., Chen H. (2015). Dual-function antibacterial surfaces for biomedical applications. Acta Biomater..

[8] Leaper D., Assadian O., Edmiston C. (2015). Approach to chronic wound infections. Br. J. Dermatol..

[9] Gorman S., Scott E. (2004). Chemical disinfectants, antiseptics and preservatives. Hugo and Russell’s Pharmaceutical Microbiology.

[10] El-Zawawy N.A., Ali S.S., Khalil M.A., Sun J., Nouh H.S. (2022). Exploring the potential of benzoic acid derived from the endophytic fungus strain Neurospora crassa SSN01 as a promising antimicrobial agent in wound healing. Microbiol. Res..

[11] Burcea A., Bănățeanu A.-M., Poalelungi C.-V., Forna N., Cumpătă C.N. (2024). Enhanced Properties and Multifaceted Applications of Polymethyl Methacrylate (PMMA) in Modern Medicine and Dentistry. Rom. J. Oral Rehabil..

[12] Wang C., Su Y., Xie J. (2024). Advances in electrospun nanofibers: Versatile materials and diverse biomedical applications. Acc. Mater. Res..

[13] Ambekar R.S., Kandasubramanian B. (2019). Advancements in nanofibers for wound dressing: A review. Eur. Polym. J..

[14] Zhu X., Zhong T., Huang R., Wan A. (2015). Preparation of hydrophilic poly (lactic acid) tissue engineering scaffold via (PLA)-(PLA-b-PEG)-(PEG) solution casting and thermal-induced surface structural transformation. J. Biomater. Sci. Polym. Ed..

[15] Mouro C., Gouveia I.C. (2024). Electrospun wound dressings with antibacterial function: A critical review of plant extract and essential oil incorporation. Crit. Rev. Biotechnol..

[16] Malikmammadov E., Tanir T.E., Kiziltay A., Hasirci V., Hasirci N. (2018). PCL and PCL-based materials in biomedical applications. J. Biomater. Sci. Polym. Ed..

[17] Kwon I.K., Kidoaki S., Matsuda T. (2005). Electrospun nano-to microfiber fabrics made of biodegradable copolyesters: Structural characteristics, mechanical properties and cell adhesion potential. Biomaterials.

[18] Wang H.B., Mullins M.E., Cregg J.M., Hurtado A., Oudega M., Trombley M.T., Gilbert R.J. (2009). Creation of highly aligned electrospun poly-L-lactic acid fibers for nerve regeneration applications. J. Neural Eng..

[19] Sivakumar M., Subadevi R., Rajendran S., Wu H.-C., Wu N.-L. (2007). Compositional effect of PVdF–PEMA blend gel polymer electrolytes for lithium polymer batteries. Eur. Polym. J..

[20] Subban R., Arof A. (2004). Plasticiser interactions with polymer and salt in PVC–LiCF3SO3–DMF electrolytes. Eur. Polym. J..

[21] Ma K., Qiu Y., Fu Y., Ni Q.-Q. (2018). Electrospun sandwich configuration nanofibers as transparent membranes for skin care drug delivery systems. J. Mater. Sci..

[22] Simões M.C.R., Cragg S.M., Barbu E., De Sousa F.B. (2019). The potential of electrospun poly (methyl methacrylate)/polycaprolactone core–sheath fibers for drug delivery applications. J. Mater. Sci..

[23] Hernandez J.L., Park J., Yao S., Blakney A.K., Nguyen H.V., Katz B.H., Jensen J.T., Woodrow K.A. (2021). Effect of tissue microenvironment on fibrous capsule formation to biomaterial-coated implants. Biomaterials.

[24] Altheide S.T. (2019). Biochemical and culture-based approaches to identification in the diagnostic microbiology laboratory. Am. Soc. Clin. Lab. Sci..

[25] Salim S.A., Kamoun E.A., Evans S., El-Moslamy S.H., El-Fakharany E.M., Elmazar M.M., Abdel-Aziz A., Abou-Saleh R., Salaheldin T.A. (2021). Mercaptopurine-loaded sandwiched tri-layered composed of electrospun polycaprolactone/poly (methyl methacrylate) nanofibrous scaffolds as anticancer carrier with antimicrobial and antibiotic features: Sandwich configuration nanofibers, release study and *in vitro* bioevaluation tests. Int. J. Nanomed..

[26] Son S.-R., Linh N.-T.B., Yang H.-M., Lee B.-T. (2013). *In vitro* and *in vivo* evaluation of electrospun PCL/PMMA fibrous scaffolds for bone regeneration. Sci. Technol. Adv. Mater..

[27] Yam W., Ismail J., Kammer H., Schmidt H., Kummerlöwe C. (1999). Polymer blends of poly (ϵ-caprolactone) and poly (vinyl methyl ether)–thermal properties and morphology. Polymer.

[28] ASTM International (2002). Standard Test Method for Tensile Properties of Thin Plastic Sheeting.

[29] Ho S.T., Hutmacher D.W. (2006). A comparison of micro CT with other techniques used in the characterization of scaffolds. Biomaterials.

[30] Young T. (1805). An essay on the cohesion of fluids. Phil. Trans. R. Soc..

[31] Xue J., Xie J., Liu W., Xia Y. (2017). Electrospun nanofibers: New concepts, materials, and applications. Acc. Chem. Res..

[32] Bismarck A., Song B., Springer J. (2000). Contact Angle Measurements on Fibers and Fiber Assemblies, Bundles, Fabrics, and Textiles. Surface and Interfacial Tension.

[33] Kenawy E.-R., Bowlin G.L., Mansfield K., Layman J., Simpson D.G., Sanders E.H., Wnek G.E. (2002). Release of tetracycline hydrochloride from electrospun poly (ethylene-co-vinylacetate), poly (lactic acid), and a blend. J. Control. Release.

[34] Skehan P., Storeng R., Scudiero D., Monks A., McMahon J., Vistica D., Warren J.T., Bokesch H., Kenney S., Boyd M.R. (1990). New colorimetric cytotoxicity assay for anticancer-drug screening. J. Natl. Cancer Inst..

[35] Hassanzadeh S., Pourmand M.R., Afshar D., Dehbashi S., Mashhadi R. (2016). TENT: A rapid DNA extraction method of *Staphylococcus aureus*. Iran. J. Public Health.

[36] Shohayeb M., El-Banna T., Elsawy L.E., El-Bouseary M.M. (2023). Panton-Valentine Leukocidin (PVL) genes may not be a reliable marker for community-acquired MRSA in the Dakahlia Governorate, Egypt. BMC Microbiol..

[37] El-Badawy M.F., Tawakol W.M., Maghrabi I.A., Mansy M.S., Shohayeb M.M., Ashour M.S. (2017). Iodometric and molecular detection of ESBL production among clinical isolates of *E. coli* fingerprinted by ERIC-PCR: The first Egyptian report declares the emergence of *E. coli* O25b-ST131clone harboring *bla*_GES_. Microb. Drug Resist..

[38] Clinical and Laboratory Standards Institute (CLSI) (2017). Performance Standards for Antimicrobial Susceptibility Testing.

[39] Goda R., Shohayeb M. (2021). Use of Pistacia lentiscus mastic for sustained—Release system of chlorocresol and benzoic acid for in vitro prevention of bacterial colonization of silicon urinary catheter. Lett. Appl. Microbiol..

[40] Roth T., Zelinger E., Kossovsky T., Borkow G. (2024). Scanning electron microscopy analysis of biofilm-encased bacteria exposed to cuprous oxide-impregnated wound dressings. Microbiol. Res..

[41] Munj H.R., Tomasko D.L. (2017). Polycaprolactone-polymethyl methacrylate electrospun blends for biomedical applications. Polym. Sci. Ser. A.

[42] Sill T.J., Von Recum H.A. (2008). Electrospinning: Applications in drug delivery and tissue engineering. Biomaterials.

[43] Piperno S., Lozzi L., Rastelli R., Passacantando M., Santucci S. (2006). PMMA nanofibers production by electrospinning. Appl. Surf. Sci..

[44] Kaper J.B., Nataro J.P., Mobley H.L. (2004). Pathogenic Escherichia coli. Nat. Rev. Microbiol..

[45] Abdelrazek E., Hezma A., El-Khodary A., Elzayat A. (2016). Spectroscopic studies and thermal properties of PCL/PMMA biopolymer blend. Egypt. J. Basic Appl. Sci..

[46] Yuan Y., Choi K., Choi S.-O., Kim J. (2018). Early stage release control of an anticancer drug by drug-polymer miscibility in a hydrophobic fiber-based drug delivery system. RSC Adv..

[47] Elashmawi I., Hakeem N. (2008). Effect of PMMA addition on characterization and morphology of PVDF. Polym. Eng. Sci..

[48] Saleh L.M., Abd Ulkadhem N.J., Kadhim Q.S., Alsaedi R.J. (2024). Polycaprolactone nanofibrous mats with electrospun polymethylmethacrylate: Synthesis and evaluation. AIP Conf. Proc..

[49] Duta L., Popescu A., Zgura I., Preda N., Mihailescu I. (2015). Wettability of nanostructured surfaces. Wetting Wettability.

[50] Negut I., Dorcioman G., Grumezescu V. (2020). Scaffolds for wound healing applications. Polymers.

[51] Linh N.T.B., Min Y.K., Song H.Y., Lee B. (2010). Fabrication of polyvinyl alcohol/gelatin nanofiber composites and evaluation of their material properties. J. Biomed. Mater. Res. Part B Appl. Biomater..

[52] Koenig M., Huang S. (1995). Biodegradable blends and composites of polycaprolactone and starch derivatives. Polymer.

[53] Cagri A., Ustunol Z., Ryser E. (2001). Antimicrobial, mechanical, and moisture barrier properties of low pH whey protein—Based edible films containing p—Aminobenzoic or sorbic acids. J. Food Sci..

[54] Hollister S.J. (2005). Porous scaffold design for tissue engineering. Nat. Mater..

[55] Boudriot U., Dersch R., Greiner A., Wendorff J.H. (2006). Electrospinning approaches toward scaffold engineering—A brief overview. Artif. Organs.

[56] Shin H.J., Lee C.H., Cho I.H., Kim Y.-J., Lee Y.-J., Kim I.A., Park K.-D., Yui N., Shin J.-W. (2006). Electrospun PLGA nanofiber scaffolds for articular cartilage reconstruction: Mechanical stability, degradation and cellular responses under mechanical stimulation *in vitro*. J. Biomater. Sci. Polym. Ed..

[57] Croisier F., Jérôme C. (2013). Chitosan-based biomaterials for tissue engineering. Eur. Polym. J..

[58] Soni M., Carabin I., Burdock G. (2005). Safety assessment of esters of p-hydroxybenzoic acid (parabens). Food Chem. Toxicol..

[59] Nowak-Lange M., Niedziałkowska K., Lisowska K. (2022). Cosmetic preservatives: Hazardous micropollutants in need of greater attention?. Int. J. Mol. Sci..

[60] Ecevit K., Barros A.A., Silva J.M., Reis R.L. (2022). Preventing microbial infections with natural phenolic compounds. Future Pharmacol..

[61] Bruinink A., Luginbuehl R. (2011). Evaluation of biocompatibility using *in vitro* methods: Interpretation and limitations. Tissue Engineering III: Cell-Surface Interactions for Tissue Culture.

[62] (2009). Biological Evaluation of Medical Devices—Part 5: Tests for In Vitro Cytotoxicity.

[63] Gruber S., Nickel A. (2023). Toxic or not toxic? The specifications of the standard ISO 10993-5 are not explicit enough to yield comparable results in the cytotoxicity assessment of an identical medical device. Front. Med. Technol..

[64] Jian L., Po A.L.W. (1993). Ciliotoxicity of methyl- and propyl-*p*-hydroxybenzoates: A dose-response and surface-response study. J. Pharm. Pharmacol..

[65] Yılmaz S., Ünal F., Yüzbaşıoğlu D. (2009). The *in vitro* genotoxicity of benzoic acid in human peripheral blood lymphocytes. Cytotechnology.

[66] Jamil M.U., Ali M.M., Firyal S., Ijaz M., Awan F., Ullah A. (2025). Comparative cytotoxic and genotoxic assessment of methylparaben and propylparaben on Allium cepa root tips by comet and Allium cepa assays. Environ. Toxicol..

[67] Torfs E., Brackman G. (2021). A perspective on the safety of parabens as preservatives in wound care products. Int. Wound J..

[68] Aldeen D., Hiramatsu K. (2004). Staphylococcus Aureus: Molecular and Clinical Aspects.

[69] Mroueh M., Issa D., Khawand J., Haraty B., Malek A., Kassaify Z., Toufeili I. (2008). Levels of benzoic and sorbic acid preservatives in commercially produced yoghurt in Lebanon. J. Food Agric. Environ..

[70] Valkova N., Lépine F.o., Valeanu L., Dupont M., Labrie L., Bisaillon J.-G., Beaudet R.j., Shareck F.o., Villemur R. (2001). Hydrolysis of 4-hydroxybenzoic acid esters (parabens) and their aerobic transformation into phenol by the resistant Enterobacter cloacae strain EM. Appl. Environ. Microbiol..

[71] Russell A., Furr J. (1996). Biocides: Mechanisms of antifungal action and fungal resistance. Sci. Prog..

[72] Neidig C., Burrell H. (1944). The esters of p-hydroxybenzene acid as preservatives. Drug Cosmet. Ind..

[73] Wang Y., Yu D.-G., Liu Y., Liu Y.-N. (2022). Progress of electrospun nanofibrous carriers for modifications to drug release profiles. J. Funct. Biomater..

